# Heterogeneity of B cell lymphopoiesis in patients with premalignant and active myeloma

**DOI:** 10.1172/jci.insight.159924

**Published:** 2023-02-08

**Authors:** Jana Jakubikova, Danka Cholujova, Gabor Beke, Teru Hideshima, Lubos Klucar, Merav Leiba, Krzysztof Jamroziak, Paul G. Richardson, Efstathios Kastritis, David M. Dorfman, Kenneth C. Anderson

**Affiliations:** 1 Dana-Farber Cancer Institute, Department of Medical Oncology, Jerome Lipper Multiple Myeloma Center, Boston, Massachusetts, USA.; 2 Department of Medicine, Harvard Medical School, Boston, Massachusetts, USA.; 3 Department of Tumor Immunology, Cancer Research Institute, Biomedical Research Center,; 4 Centre for Advanced Materials Application, and; 5 Institute of Molecular Biology, Slovak Academy of Sciences, Bratislava, Slovakia.; 6 Department of Hematology, Samson Assuta Ashdod University Hospital, Ashdod, Israel.; 7 Faculty of Health Sciences, Ben Gurion University of the Negev, Beer Sheva, Israel.; 8 Department of Hematology, Transplantation and Internal Medicine, Medical University of Warsaw, Warsaw, Poland.; 9 Department of Clinical Therapeutics, School of Medicine, National and Kapodistrian University of Athens, Athens, Greece.; 10 Department of Pathology, Brigham and Women’s Hospital, Boston, Massachusetts, USA.

**Keywords:** Hematology, Oncology, Bone marrow, Clonal selection

## Abstract

To better characterize the heterogeneity of multiple myeloma (MM), we profiled plasma cells (PCs) and their B cell lymphopoiesis in the BM samples from patients with monoclonal gammopathy of undetermined significance, smoldering MM, and active MM by mass cytometry (CyTOF) analysis. Characterization of intra- and interneoplastic heterogeneity of malignant plasmablasts and PCs revealed overexpression of the MM SET domain (MMSET), Notch-1, and CD47. Variations in upregulation of B cell signaling regulators (IFN regulatory factor 4 [IRF-4], CXCR4, B cell lymphoma 6 [Bcl-6], c-Myc, myeloid differentiation primary response protein 88 [MYD88], and spliced X box-binding protein 1 [sXBP-1]) and aberrant markers (CD319, CD269, CD200, CD117, CD56, and CD28) were associated with different clinical outcomes in clonal PC subsets. In addition, prognosis was related to heterogeneity in subclonal expression of stemness markers, including neuroepithelial stem cell protein (Nestin), SRY-box transcription factor 2 (Sox2), Krüppel-like factor 4 (KLF-4), and Nanog. Furthermore, we have defined significantly elevated levels of MMSET, MYD88, c-Myc, CD243, Notch-1, and CD47 from hematopoietic stem cells to PCs in myeloma B cell lymphopoiesis, noted even in premalignant conditions, with variably modulated expression of B cell development regulators, including IRF-4, Bcl-2, Bcl-6, and sXBP-1; aberrant PC markers (such as CD52, CD44, CD200, CD81, CD269, CD117, and CXCR4); and stemness-controlling regulators, including Nanog, KLF-4, octamer-binding transcription factor 3/4 (Oct3/4), Sox2, and retinoic acid receptor α2 (RARα2). This study provides the rationale for precise molecular profiling of patients with MM by CyTOF technology to define disease heterogeneity and prognosis.

## Graphical abstract

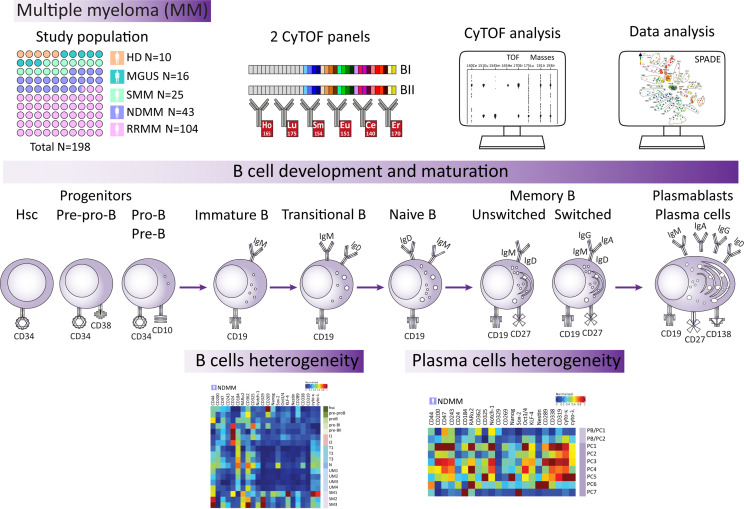

## Introduction

Multiple myeloma (MM), the second most common hematologic malignancy worldwide, represents a prototypical disease model for the study of tumor heterogeneity due to the high frequency of intraclonal diversity within malignant clones of plasma cells (PCs) in the BM. The clinical manifestations of the disease are associated with high levels of monoclonal Ig protein in serum and/or urine, BM plasmacytosis, and CRAB features, namely hypercalcemia, renal insufficiency, anemia, and/or bone disease (1). Even without CRAB features, the extent of BM plasmacytosis, bone disease on sensitive imaging, and a κ/λ ratio are MM-defining events. The premalignant spectrum of MM includes asymptomatic stages known as monoclonal gammopathy of undetermined significance (MGUS) with low levels of monoclonal BM PCs, monoclonal protein, and rates of progression, as well as smoldering MM (SMM) with a higher proportion of BM PCs, monoclonal protein, and progression to active BM (2). An improved understanding of the MM pathophysiology has derived the identification, validation, and clinical translation of several classes of treatments, including immunomodulatory drugs (thalidomide, lenalidomide, and pomalidomide); proteasome inhibitors (bortezomib, carfilzomib, and ixazomib); monoclonal Abs (daratumumab, elotuzumab, and isatuximab); a histone deacetylase inhibitor (panobinostat); a nuclear transport inhibitor (selinexor); an immunotoxin (belantomab mafodotin); and idecabtagene vicleucel chimeric antigen receptor (CAR) T cell therapy, which have transformed the treatment paradigm and markedly improved patient outcome (3, 4). Despite this remarkable progress, disease frequently relapses and novel treatments are urgently needed.

Ab-secreting PCs develop from hematopoietic stem cells (hscs) in several rounds of differentiation stages of B cell development, from B cell precursors to naive mature B lymphocytes in the BM and followed by maturation to the memory/effector B cells in the secondary lymphoid organs. Different stages of B cell development are accompanied by multiple changes of the cell immunophenotype and regulators of differentiation (5). Moreover, normal PC differentiation is tightly controlled by the coordinated regulation of transcriptional factors, including IFN regulatory factor 4 (IRF-4), B cell lymphoma 6 (Bcl-6), B lymphocyte-induced maturation protein 1 (Blimp-1), paired box gene 5 (Pax-5), and X box-binding protein 1 (6, 7). Therefore, insight into B cell lymphopoiesis is essential for understanding the pathogenesis of MM. Myelomagenesis results from a host of primary genetic abnormalities, including chromosomal translocations involving the Ig heavy chain genes and aneuploidy, as well as secondary genetic alterations such as copy number variants, oncogenic mutations, and epigenetic alterations (8, 9). Moreover, numerous signaling pathways are constitutively activated and/or downregulated in MM such as PI3K, NF-κB, RAS/RAF/MAPK, JAK/STAT, and Myc. These alterations are associated with hallmarks of MM, including abnormal PC differentiation, deregulation of cell cycle, decreased apoptosis, and increased MM cell growth and survival (6). In addition, the molecular events acquired during MM progression support the concept of branching clonal evolution (10, 11). Ongoing studies are further characterizing deregulation of this coordinated network of genetic alterations and signaling pathways, as well as intraclonal dynamics, as leading to MM transformation in the BM milieu.

In this study, we report a comprehensive analysis of PC heterogeneity within the B cell development of the BM microenvironment in 16 patients with MGUS, 25 patients with SMM, 43 newly diagnosed patients with MM (NDMM), and 104 patients with relapsed or relapsed/refractory MM (RRMM), as well as 10 healthy donors (HDs), using data-driven high-dimensional mass cytometry (CyTOF) analysis. Our pipeline has been designed for profiling PCs and maturation stages of B cell lymphopoiesis/B lineage differentiation during MM evolution and progression. Characterization of malignant plasmablast (PB) and PC heterogeneity revealed overexpression of MMSET, Notch-1, and CD47, with variations in upregulation of B cell signaling regulators (IRF-4, Bcl-6, c-Myc, CXCR4, myeloid differentiation primary response protein 88 [MYD88], and spliced XBP-1 [sXBP-1]), PC aberrant markers (CD319, CD269, CD200, CD117, CD56, and CD28), and stemness markers, including neuroepithelial stem cell protein (Nestin), SRY-box transcription factor 2 (Sox2), Krüppel-like factor 4 (KLF-4), and Nanog, that were associated with different clinical outcomes in clonal PC subsets. In addition, various immunophenotypic profiles and modulation of signaling in B cell lymphopoiesis were defined, even in premalignant myeloma stages, confirming clonal hematopoiesis. Our in-depth molecular characterization of PCs in the B cell ecosystem at various stages of MM demonstrates the utility of CyTOF technology for defining disease heterogeneity and prognosis in patients with MM.

## Results

### Study pipeline of high-dimensional single-cell profiling of MM cohort by CyTOF.

**Figure 1:**
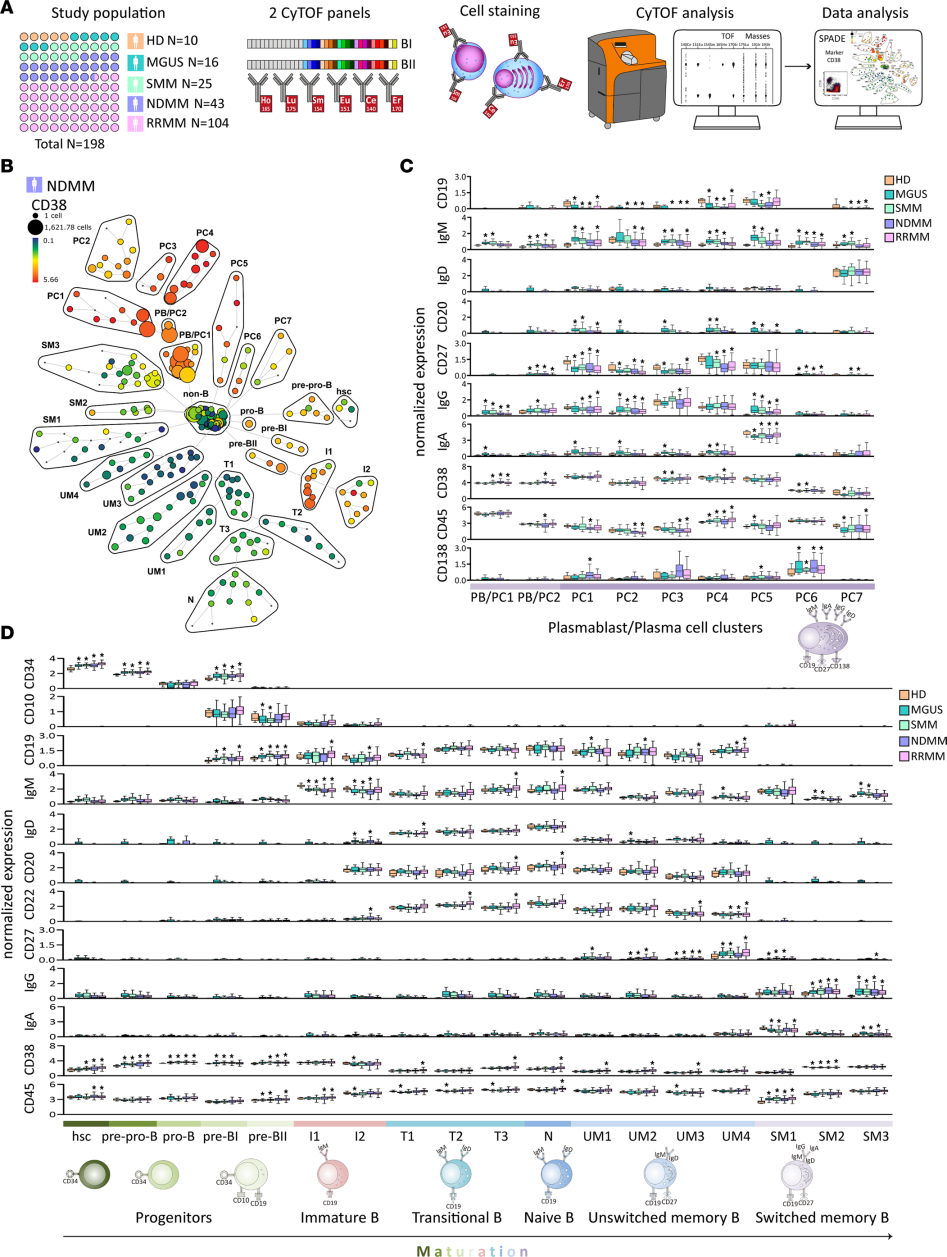
High-dimensional single-cell profiling of B cell lymphopoiesis in MM by CyTOF analysis. (**A**) Schema of experimental design used in this study. (**B**) SPADE analysis of B lymphoid cell subsets in representative BM sample of NDMM patient. Each node of the SPADE tree is colored for the median expression of CD38, with the size of each node correlated to amount of the cells. (**C**) Box plots of normalized median expression of B cell markers (CD34, CD10, CD19, IgM, IgD, CD20, CD22, CD27, IgG, IgA, CD38, and CD45) in MGUS (*n* = 16), SMM (*n* = 25), NDMM (*n* = 43), and RRMM (*n* = 104) versus HDs (*n* = 10). The maturation spectrum from hscs to Ig-switched memory B cells is defined by color code. Each box represents 0.25–0.75 percentile of median expression, with whiskers calculated by Tukey’s method. Multiple clusters in some B cell subsets are identified. Significant differences between MGUS, SMM, NDMM, and RRMM versus HDs are defined by Dunn’s multiple comparison test after the Kruskal-Wallis 1-way ANOVA by ranks, *P value < 0.05. (**D**) Box plots of normalized median expression of B cell markers (CD19, IgM, IgD, CD20, CD27, IgG, IgA, CD38, CD45, and CD138) in MGUS (*n* = 16), SMM (*n* = 25), NDMM (*n* = 43), and RRMM (*n* = 104) versus HDs (*n* = 10) in PB/PC clusters (PB/PC1–2) and PC clusters (PC1–7) defined by color code (bottom). Each box represents 0.25–0.75 percentile of median expression, with whiskers calculated by Tukey’s method. Significant differences identified between MGUS, SMM, NDMM, and RRMM versus HDs are defined by Dunn’s multiple comparison test after the Kruskal-Wallis 1-way ANOVA by ranks, *P value < 0.05.

To better understand the heterogeneity of MM, we performed large-scale CyTOF analysis in a cohort of 188 BM samples from patients with MM compared with 10 age-matched HDs. The clinical characteristics of our study cohort are shown in Supplemental Table 1; supplemental material available online with this article; https://doi.org/10.1172/jci.insight.159924DS1 We designed a pipeline for deep characterization of malignant PC within the B cell lymphopoiesis in patients with MGUS (*n* = 16), SMM (*n* = 25), NDMM (*n* = 43), and RRMM (*n* = 104) (Figure 1A). We focused on profiling of PC heterogeneity within patients and between these groups based on molecular perturbations of transcriptional factors and signaling regulators (BI panel), as well as stemness-controlling and aberrant PC markers (BII panel). In addition, both panels profiled B cell lymphopoiesis in MM. To characterize PC clonal cells within myeloma B cell lymphopoiesis, we supplemented out 13 B cell specific markers in each B panel (BI and BII) with 20 signaling markers, either intracellular or activation cell surface molecules, as well as cytoplasmic κ (cyto κ) and λ (cyto λ) light chain for clonality assessment. In our CyTOF panels, the Abs were conjugated with rare stable earth elemental metals (lanthanides) followed by determination of metal content of labeled Abs with greater than 100 metal atoms per Ab. The specificity and efficacy of these Abs were evaluated using negative and positive controls, and titrations defined optimal concentrations for use in CyTOF analysis (Supplemental Table 2). Briefly, BM cells isolated from these cohorts (either presorted or not for BI and BII panels) were stained in parallel with these BI and BII panels and analyzed by CyTOF technology (Supplemental Figure 1) followed by comprehensive bioinformatics and statistical data analysis (Figure 1A; and Supplemental Table 3).

### Mapping myeloma B cell lymphopoiesis.

To profile myeloma B cell lymphopoiesis in our cohorts, we used 13 B cell stage-specific markers (CD10, CD19, CD20, CD22, CD27, CD34, CD38, CD45, CD138, IgA, IgD, IgG, and IgM) in BI and BII panels to profile B lymphoid maturation stages by spanning-tree progression analysis of density-normalized events (SPADE) clustering analysis. A representative NDMM BM showing B lymphoid subsets is seen in CD38 expression (Figure 1B) and other B cell clustering markers (Supplemental Figure 2). To ensure that sorting to remove granulocytes (CD15^+^ cells) did not impact analysis using BI and BII panels, we compared 5 sorted versus unsorted HD BM samples. Correlation analyses between the same sorted and unsorted samples showed a high degree of reproducibility and accuracy for CyTOF analysis with *R*^2^ = 0.98 (Supplemental Figure 3). In addition, a high correlation between both B panels (BI and BII) was confirmed for all 13 B cell markers (Supplemental Figure 4). As MM is a tumor of Ab-producing PCs, precursors of PBs and PCs were identified by increased expression of CD38, decreased expression of CD45, and heterogenous (^het^) expression of CD19, CD20, CD27, and CD138 with surface membrane Igs (mIgs), such as IgA, IgG, and IgD (Figure 1C). Moreover, analysis of negative and positive coexpression of 13 B cell clustering markers by SPADE analysis was used to evaluate maturation of B lymphoid lineage from early B cell progenitors to further profiled B cell maturation by identifying clusters of immature, transitional, and naive B cells and clusters of memory, either unswitched or switched, B cells (Figure 1D). Taken together, these data demonstrate various immunophenotypic aberrancies at different stages of B cell lymphopoiesis in MM.

### Phenotypic and signaling aberrations in myeloma B cell progenitors.

**Figure 2:**
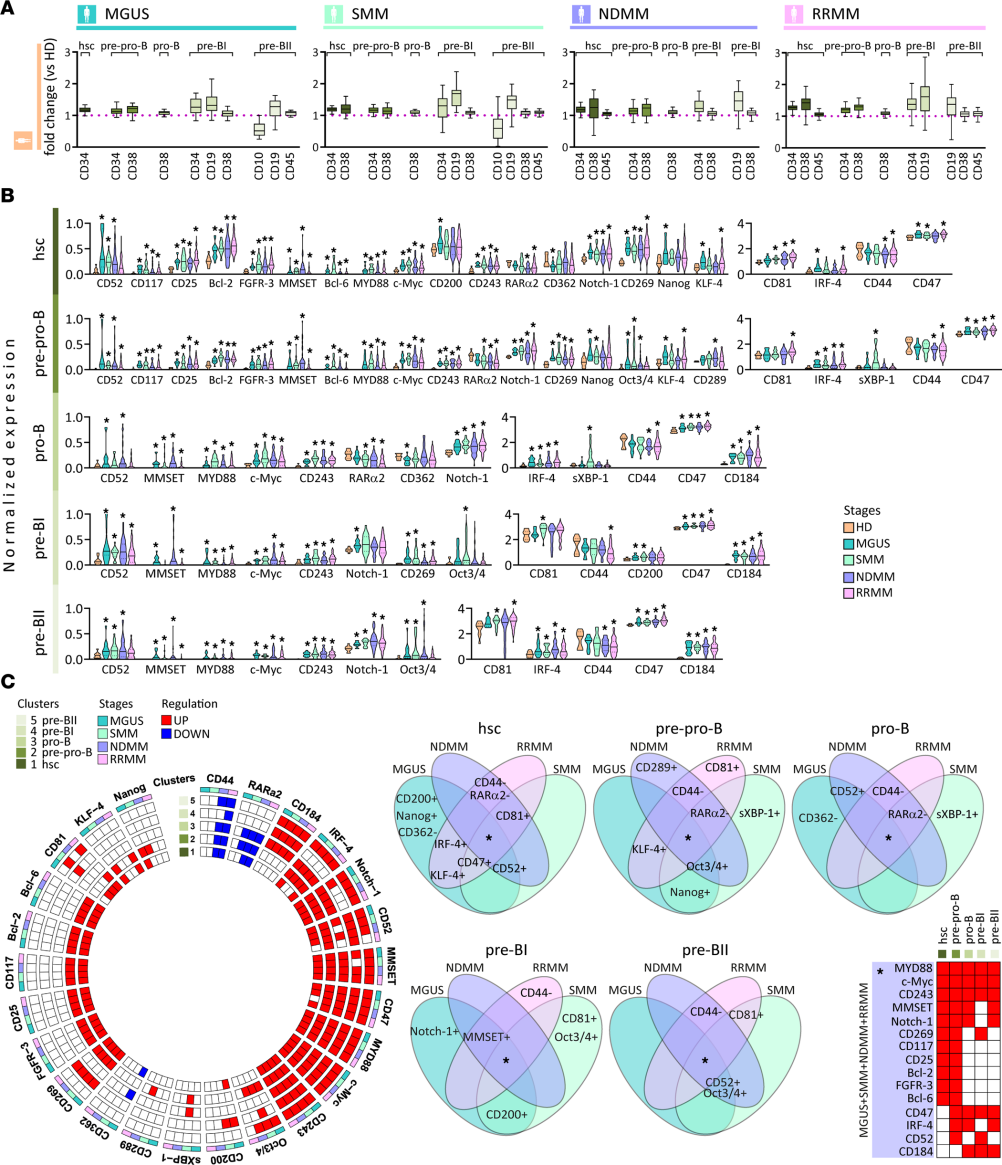
Phenotypic and signaling changes in B cell precursors of MM. (**A**) Notched boxes represent the 25th and 75th percentile values with Tukey whiskers of the ratio of statistically significant median expression for the indicated clustering markers calculated by Mann-Whitney *U* test, *P* value < 0.05. (**B**) Violin plots show statistically significant normalized median expression of signaling markers on B cell precursors (hsc, pre-pro-B, pro-B, pre-BI, and pre-BII) in MGUS (*n* = 16), SMM (*n* = 25), NDMM (*n* = 43), and RRMM (*n* = 104) versus HDs (*n* = 10), represented by color codes. Significant differences were defined by Dunn’s multiple comparison test after the Kruskal-Wallis 1-way ANOVA by ranks, *P value < 0.05. (**C**) Circle diagrams show schematic summary of statistically significant normalized median expression of signaling markers either downregulated (blue rectangle) or upregulated (red rectangle) within B cell precursors (hsc, pre-pro-B, pro-B, pre-BI, and pre-BII) in MGUS, SMM, NDMM, and RRMM versus HDs by Dunn’s multiple comparison test after the Kruskal-Wallis 1-way ANOVA by ranks, with *P* value < 0.05. Venn diagrams show 15 intersections among 4 MM disease stages — MGUS, SMM, NDMM, and RRMM. Each intersection shows joint expression of statistically significant signaling markers either downregulated (–) or upregulated (+) within B cell progenies (hsc, pre-pro-B, pro-B, pre-BI, and pre-BII) in MGUS, SMM, NDMM, and RRMM compared with HDs.

To examine maturation of myeloma B lymphoid lineage, we first evaluated B cell precursors/progenitors from CD34^+^, CD38^lo^, and CD45^lo^ hscs to pre-pro-B (CD34^+^, CD38^het^, and CD45^lo^), pro-B (CD34^lo^, CD38^+^, and CD45^lo^), pre-BI (CD34^het^, CD10^+^, and CD19^lo^), and pre-BII (CD34^–^, CD10^lo^, CD19^lo^, and IgM^lo^) cells. Comparing MM disease stages and clustering differences with HDs on early B cell progenies (hsc, pre-pro-B, and pro-B), we observed a significant increase in expression of CD34 and CD38 in MGUS, SMM, NDMM, and RRMM (Figure 2A). Upregulation of CD19, CD38, and CD45 was observed on both pre-BI and pre-BII cells in MGUS, SMM, NDMM, and RRMM versus HDs, whereas the downregulation of CD10 was noted only on pre-BII cells in MGUS and SMM (Figure 2A). We next examined signaling in early MM B cell lymphopoiesis and observed significant overexpression of CD52, MMSET, MYD88, c-Myc, CD243, Notch-1, and CD47 in B cell progenitors (Figure 2B) in all MM stages, whereas downregulated expression of CD44 was detected in active MM stages. Significant modulation of CD117, CD25, Bcl-2, fibroblast growth factor receptor 3 (FGFR3), Bcl-6, retinoic acid receptor α2 (RARα2), CD269, Nanog, KLF-4, CD81, and IRF-4 expression was defined in both hsc and pre-pro-B cells of MM, as well as differences in expression of CD200, CD362 (hsc), octamer-binding transcription factor 3/4 (Oct3/4), CD289, and sXBP-1 (pre-pro-B). Upregulation of CXCR4 (CD184) was observed from late pro-B cells in all MM stages. Significant differences were noted among pro-B (RARα2, CD362, IRF-4, and sXBP1), pre-BI (CD269, Oct3/4, CD81, and CD200) and pre-BII (Oct3/4, CD81, and IRF-4). Summary circle and Venn diagrams show upregulated B cell signaling regulators (including MYD88, MMSET, c-Myc, Notch-1, CXCR4, IRF-4, Bcl-6, and Bcl-2), ATP-binding cassette (ABC) transporter CD243, and PC aberrant markers (such as CD47, CD52, CD269, CD117, and CD25) in B cell precursors/progenitors in all MM stages (Figure 2C). In addition, an increase in CD200 (hsc and pre-BI), Nanog (hsc and pre-pro-B), sXBP-1 (pre-pro-B and pro-B), Oct3/4 (pre-BI), and CD81 (pre-BI) and a decrease in CD362 (hsc and pro-B) are noted only in MGUS or SMM, showing clonal hematopoiesis extending to premalignant stages of myeloma.

### Phenotypic and signaling changes in myeloma transition of immature to mature B cells.

**Figure 3:**
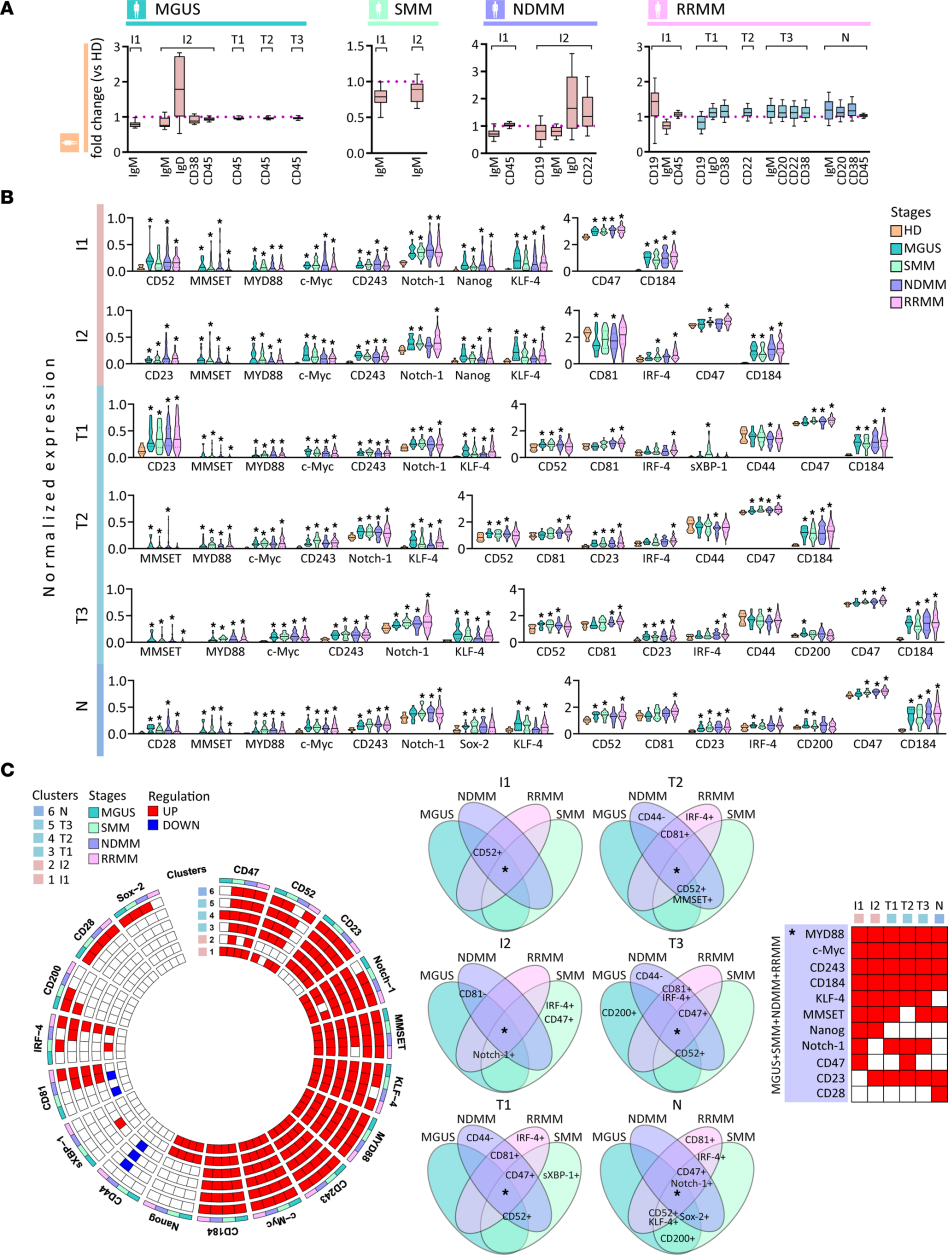
Phenotypic and signaling aberrations from immature to naive B cells of MM. (**A**) Notched boxes represent the 25th and 75th percentile values with Tukey whiskers of the ratio of statistically significant median expression for the indicated clustering markers calculated by Mann-Whitney *U* test, *P* value < 0.05. (**B**) Violin plots show statistically significant normalized median expression of signaling markers on immature (I1–2 clusters), transitional (T1–3 clusters), and naive (N) B cell clusters in MGUS (*n* = 16), SMM (*n* = 25), NDMM (*n* = 43), and RRMM (*n* = 104) versus HDs (*n* = 10), represented by color codes. Significant differences were defined by Dunn’s multiple comparison test after the Kruskal-Wallis 1-way ANOVA by ranks, *P value < 0.05. (**C**) Circle diagrams show schematic summary of statistically significant normalized median expression of signaling markers either downregulated (blue rectangle) or upregulated (red rectangle) within immature (I1–2), transitional (T1–3), and naive (N) B cells in MGUS, SMM, NDMM, and RRMM versus HDs by Dunn’s multiple comparison test after the Kruskal-Wallis 1-way ANOVA by ranks, with *P* value < 0.05. Venn diagrams show 15 intersections among MGUS, SMM, NDMM, and RRMM, and each intersection shows joint expression of statistically significant signaling markers either downregulated (–) or upregulated (+) within immature (I1–2), transitional (T1–3), and naive (N) B cells in MGUS, SMM, NDMM, and RRMM versus HDs.

We further profiled B cell maturation by identifying clusters of immature (I1–2 clusters; CD10^lo^, CD19^+^, IgM^+^, IgD^–/lo^, CD20^–/+^, and CD27^–^), transitional (T1–3 clusters; CD10^–^, CD19^+^, IgM^+^, IgD^lo^, CD20^+^, CD22^+^, and CD27^–^), and naive (N; CD19^+^, IgM^+^, IgD^+^, CD20^+^, CD22^+^, and CD27^–^) B cells. In-depth analysis of immature clusters showed decreased expression of IgM and increased expression of IgD in MGUS, SMM, NDMM, and RRMM compared with HDs. Although no significant changes were revealed in clusters from transitional to naive B cells in MGUS, SMM, and NDMM, substantial upregulation in CD20, CD22, IgD, IgM, and CD38 expression was observed in RRMM (Figure 3A). In signaling profiling of B lymphoid lineage, significant upregulation of MMSET, MYD88, c-Myc, CD243, Notch-1, KLF-4, CD47, and CXCR4 was observed from immature to naive B cells in all MM stages. In addition, we detected increased expression of CD52, CD23, and IRF-4 and modulation in expression of CD81 (I2) in the majority of clusters from immature B cells to naive B cells in our MM cohort. Upregulation of Nanog (I1–2), sXBP-1 (T1), CD200 (T3 and N), CD28, and Sox2 (N), as well as downregulation of CD44 (T1–3), was observed (Figure 3B). Mostly upregulated B cell signaling regulators (such as MMSET, MYD88, c-Myc, CXCR4, and Notch-1), stem cell marker KLF-4, ABC transporter CD243, and PC aberrant markers (CD47 and CD52) were noted from immature to naive B cells in all MM stages with upregulation of sXBP-1 (T1) and CD200 (T3 and N) only in MGUS or SMM (Figure 3C), showing signaling perturbations in both premalignant and active MM stages.

### Phenotypic and signaling abnormalities in myeloma memory B cells.

**Figure 4:**
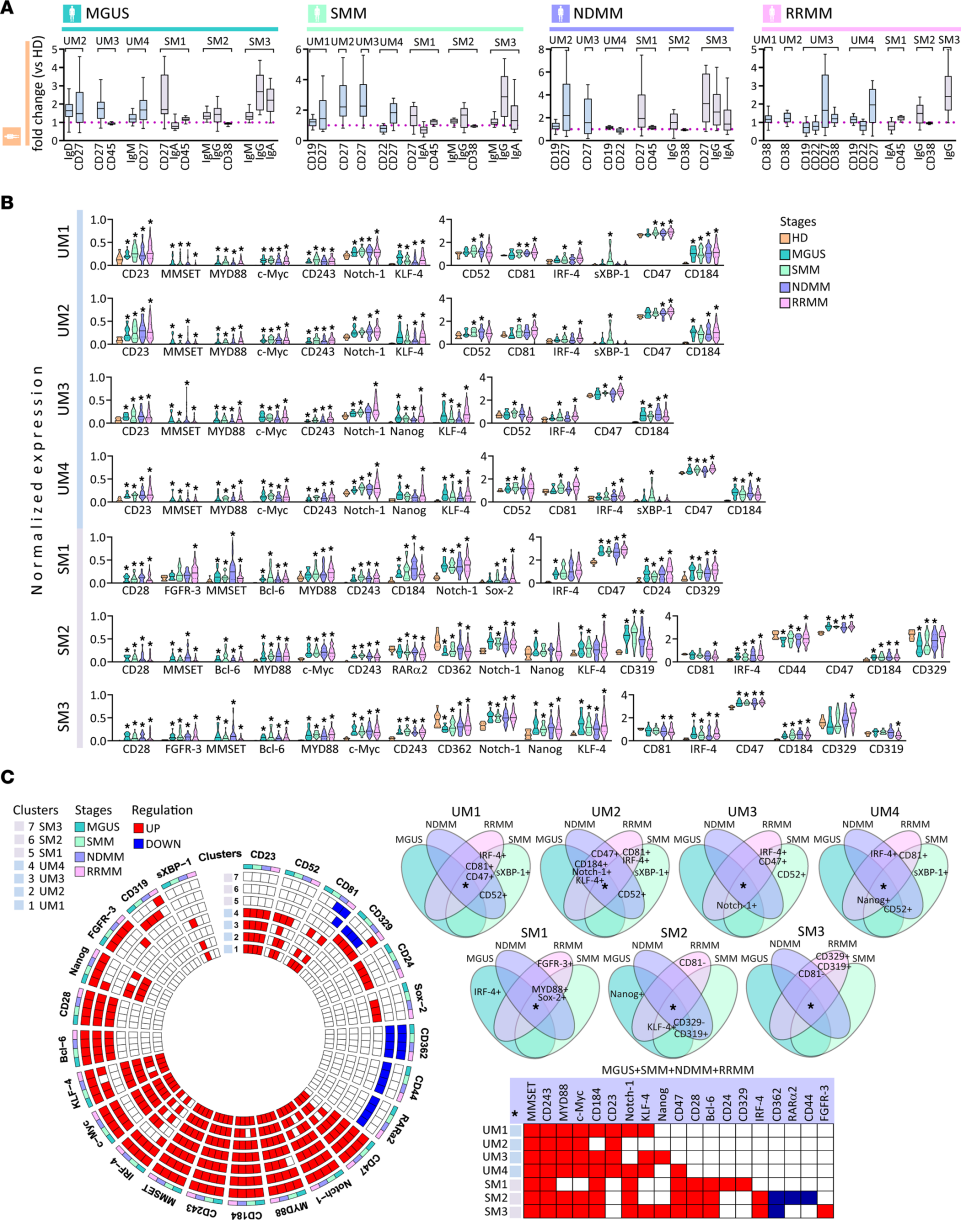
Phenotypic and signaling changes in memory B cells of MM. (**A**) Notched boxes represent the 25th and 75th percentile values with Tukey whiskers of the ratio of statistically significant median expression for the indicated clustering markers calculated by the Mann-Whitney *U* test, *P* value < 0.05. (**B**) Violin plots show statistically significant normalized median expression of signaling markers on unswitched (UM1–4) and switched (SM1–3) clusters of memory B cells in MGUS (*n* = 16), SMM (*n* = 25), NDMM (*n* = 43), and RRMM (*n* = 104) versus HDs (*n* = 10), represented by color codes. Significant differences were defined by Dunn’s multiple comparison test after the Kruskal-Wallis 1-way ANOVA by ranks, *P value < 0.05. (**C**) Circle diagrams show schematic summary of statistically significant normalized median expression of signaling markers either downregulated (blue rectangle) or upregulated (red rectangle) within memory B cells from unswitched (UM1–4) to switched (SM1–3) memory B cells in MGUS, SMM, NDMM, and RRMM versus HDs by Dunn’s multiple comparison test after the Kruskal-Wallis 1-way ANOVA by ranks, with *P* value < 0.05. Venn diagrams show 15 intersections among MGUS, SMM, NDMM, and RRMM, and each intersection shows joint expression of statistically significant signaling markers either downregulated (–) or upregulated (+) within memory B cells from unswitched (UM1–4) to switched (SM1–3) memory B cells in MGUS, SMM, NDMM, and RRMM compared with HDs.

To evaluate myeloma memory B cells, we identified clusters of memory B cells that were either unswitched (UM1–4 clusters; CD19^+^, IgM^+^, IgD^het^, CD20^+^, CD22^+^, and CD27^+^) or switched (SM1–3 clusters; IgM^+^, IgD^–^, CD27^het^, IgG^+^, and IgA^+^). Significant differences were identified in CD19, CD22, and CD27 expression profile on unswitched memory B cells (UM1–4) in SMM, NDMM, and RRMM versus HDs, whereas MGUS differed only in increases IgD and IgM expression. Similarly, significant modulation (*P* value < 0.05) of CD27, IgM, IgG, and IgA on switched memory B cells (SM1–3) of MGUS, SMM, NDMM, and RRMM was observed (Figure 4A). Similar to earlier B cell lymphoid stages, signaling profile of both memory B cells showed increased expression of MMSET, MYD88, c-Myc, CD243, Notch-1, CD47, and CXCR4 proteins in all MM stages. In addition, unswitched memory B cells showed increased expression of CD23, KLF-4, CD52, and IRF-4 and differed in overexpression of Nanog, sXBP-1, and CD81. Furthermore, all switched memory B cells from the MM cohort showed increased expression of CD28, Bcl-6, IRF-4, and CD329, while switched memory B cell clusters differed in the expression of FGFR3 (SM1, SM3); Sox2 and CD24 (SM1); RARα2 and CD44 (SM2); and CD362, Nanog, KLF-4, CD319, and CD81 (SM2–3) expression (Figure 4B). Similar to earlier B maturation stages, the summary circle and Venn diagrams show mostly upregulated B cell signaling regulators (including MMSET, MYD88, c-Myc, CXCR4, and Notch-1), stem cell marker KLF-4, ABC transporter CD243, and PC aberrant marker CD47 in all stages of MM, with upregulated sXBP-1 only in SMM (Figure 4C). Overall, various immunophenotypic aberrancies with signaling variations are present at the earlier stages of B cell lymphopoiesis in MM.

### In-depth immunophenotyping and distribution of PCs.

MM is characterized by clonal proliferation of malignant PCs; therefore, we focused on profiling of PBs (PB/PC1–2 clusters; CD20^–^, CD27^het^, CD38^+^, CD45^+^, and CD138^–^) as precursor of PCs and mature PCs (PC1–7 clusters; CD19^het^, CD20^het^, CD27^het^, CD38^+^, CD45^lo^, and CD138^+^). Interestingly, the highest number of clusters PC1–7 was observed in PCs. Comparing MM to HDs, significant upregulation of CD27, IgG, and IgM was observed on PB (PB/PC1–2) clusters (Figure 1C and Supplemental Figure 5). In contrast, on PC clusters, a significant increase in expression of B cell marker CD20 and surface mIgs (IgM, IgA, and IgM) was observed in MGUS and SMM, whereas downregulation of B cell markers CD19 and CD27 (also in MGUS and SMM), mIgs, CD38, and CD45 was evident in NDMM and RRMM (Figure 1C and Supplemental Figure 5). Therefore, various immunophenotypic aberrancies are present not only on PC clusters, but also at earlier stages of B cell lymphopoiesis.

**Figure 5:**
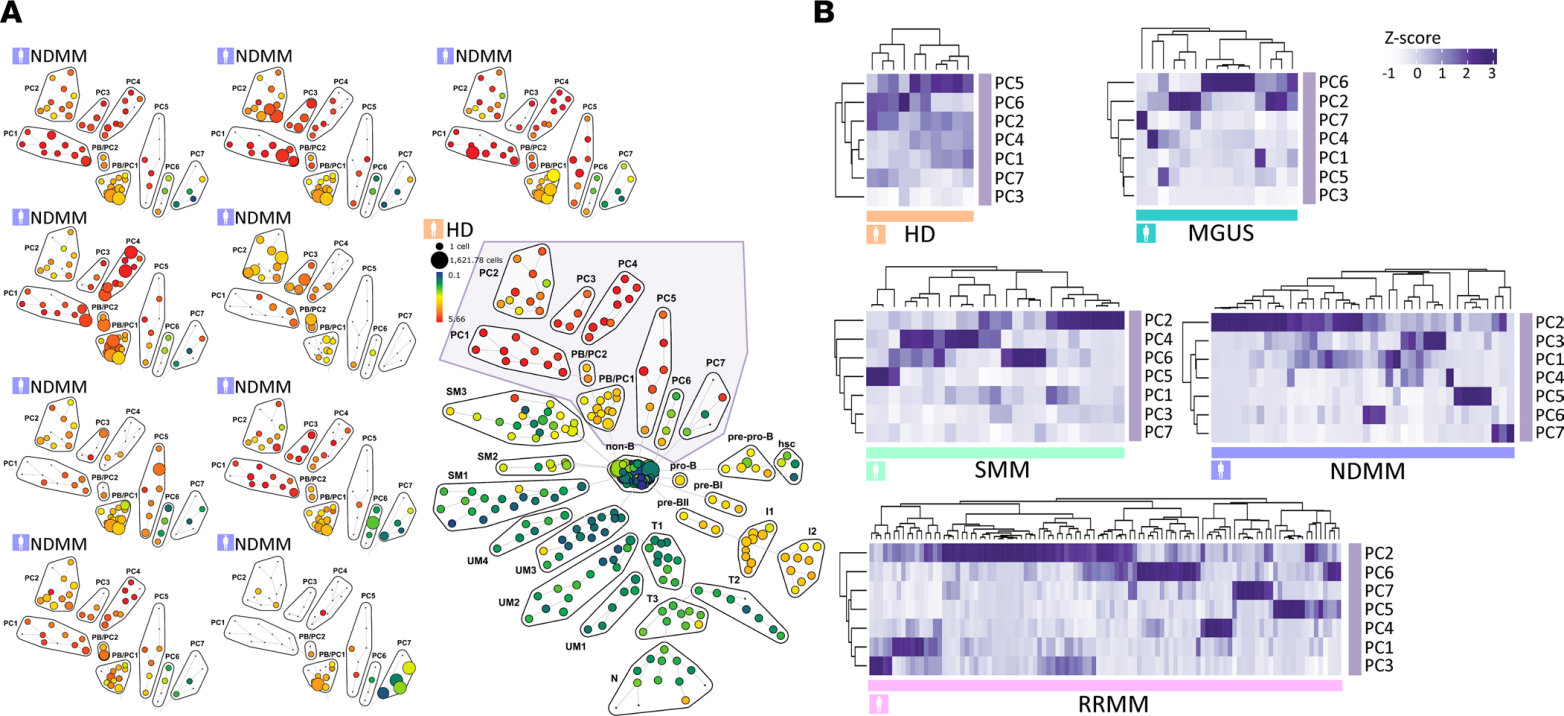
Heterogeneous PC clonal clusters in MM by CyTOF analysis. (**A**) SPADE analysis of clonal PC clusters in individual BM samples of 9 ND patients with MM show different patterns of PB/PC1–2 and PC1–7 clonal clusters compared with HDs (frame). Each node of the SPADE tree is colored for the median expression of CD38, and the size of each node is correlated to the number of cells. (**B**) *Z* score–clustered heatmaps show normalized cell frequency of PC1–7 clonal clusters in HDs (*n* = 10), MGUS (*n* = 16), SMM (*n* = 25), NDMM (*n* = 43), and RRMM (*n* = 104).

We next assessed variations in the quantity of PC clusters. Representative SPADE analyses of PC clonal clusters in individual BM samples of 9 ND patients with MM depicted differences in abundance of PB/PC1–2 and PC1–7 clonal clusters compared with HDs (frame; Figure 5A). Unsupervised *z* score–clustered heatmaps according to MM disease stage showed: the PC2 cluster dominated in SMM, NDMM, and RRMM; PC2 was associated with PC1 and PC3 in NDMM; and the clustering sequence of PC in RRMM stage appears similar to MGUS (Figure 5B). Overall, these results identify different PC clusters with variations in phenotype and abundance, which may correspond to MM subclones.

### In-depth characterization of PC heterogeneity.

**Figure 6:**
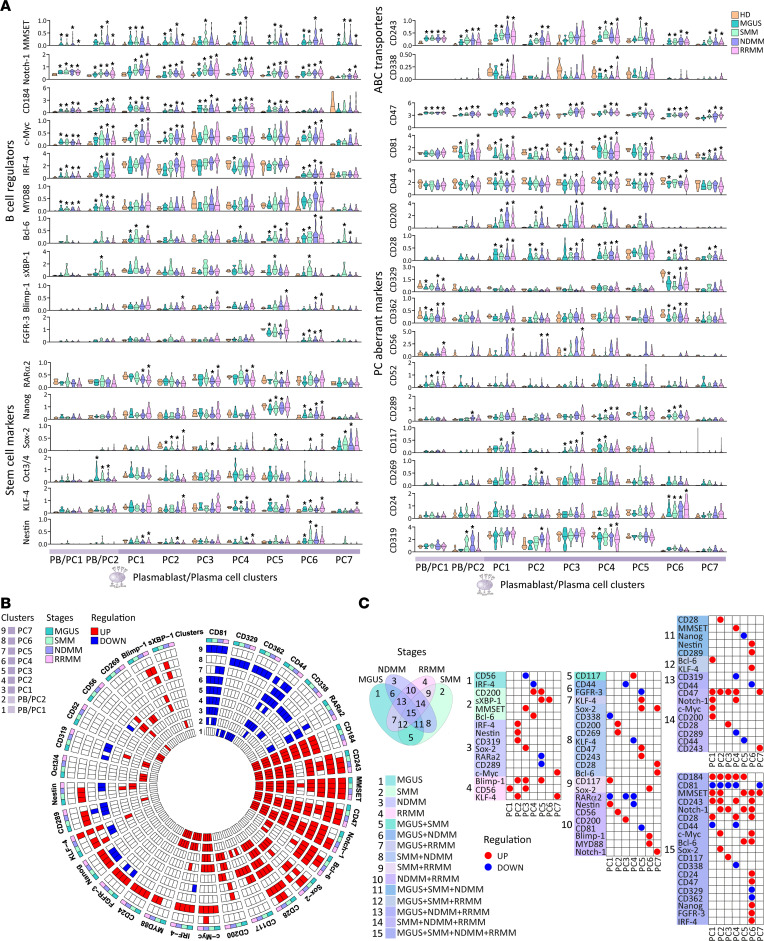
Inter- and intratumor signaling heterogeneity of clonal PCs in MM by CyTOF analysis. (**A**) Violin plots show normalized median expression for B cell regulators (MMSET, Notch-1, CD184, c-Myc, IRF-4, MYD88, Bcl-6, sXBP-1, Blimp-1, and FGFR3), stem cell markers (RARα2, Nanog, Sox2, Oct3/4, KLF-4, and Nestin), ABC transporters (CD243 and CD338), and PC aberrant markers (CD47, CD81, CD44, CD200, CD28, CD329, CD362, CD56, CD52, CD289, CD117, CD269, CD24, and CD319) in MGUS (*n* = 16), SMM (*n* = 25), NDMM (*n* = 43), and RRMM (*n* = 104) versus HDs (*n* = 10) in PB/PC clusters (PB/PC1–2) and PC clusters (PC1–7) defined by color code (bottom). Significant differences between MGUS, SMM, NDMM, and RRMM versus HDs are defined by Dunn’s multiple comparison test after the Kruskal-Wallis 1-way ANOVA by ranks, with *P value < 0.05. (**B**) Circle diagram shows schematic summary of statistically significant normalized expression of signaling markers either downregulated (blue rectangle) or upregulated (red rectangle) within PB/PC clusters (PB/PC1–2) and PC clusters (PC1–7) in MGUS, SMM, NDMM, and RRMM versus HDs by Dunn’s multiple comparison test after the Kruskal-Wallis 1-way ANOVA by ranks, with *P* value < 0.05. (**C**) Venn diagram shows 15 intersections among 4 MM disease stages — MGUS, SMM, NDMM, and RRMM. Each intersection shows statistically significant downregulation (blue circle in table) or upregulation (red circle in table) within PC clonal clusters (PC1–7) in MGUS, SMM, NDMM, and RRMM versus HDs.

Examination of intra- and interneoplastic heterogeneity of PC signaling in patients versus HDs revealed overexpressed MMSET, also known as Wolf-Hirschhorn syndrome candidate 1 (WHSC1), CD47, and Notch-1 across both PB/PC1–2 and PC1–7 clusters (Figure 6A, summarized circle diagram in Figure 6B, and Supplemental Figure 6). Profiling of PBs showed constitutive upregulation of CD184 (CXCR4), c-Myc, IRF-4, MYD88, and CD243 in MGUS, SMM, NDMM, and RRMM. Significant differences between PB/PC clusters were defined by signaling signature: PB/PC1 (CD52^hi^, CD56^hi^, CD362^lo^, and CD329^lo^) and PB/PC2 (CD81^lo^, CD44^lo^, CD289^hi^, CD319^hi^, sXBP-1^hi^, and Oct3/4^hi^). Compared to PB/PC clusters, PC1–5 clusters overexpressed MMSET, CD47, Notch-1, CXCR4, and CD200 and decreased CD81 activation marker associated with upregulation of CD243 (not detected in PC3), CD28 (not in PC5), and downregulation of CD44 and RARα2 (not detected only in PC2) expression compared with HDs. A potentially unique phenotype of each PC cluster was identified: PC1 (c-Myc^hi^, Bcl-6^hi^, CD56^hi^, Nestin^lo^, CD117^hi^, and CD338^lo^); PC2 (c-Myc^hi^, IRF-4^hi^, Sox2^lo^, KLF-4^hi^, CD56^hi^, Nestin^lo^, Blimp-1^hi^, CD319^hi^, and CD269^hi^); PC3 (Sox2^lo^, CD56^hi^, Blimp-1^hi^, and CD117^hi^); PC4 (IRF-4^lo^, Bcl-6^hi^, KLF-4^lo^, CD289^lo^, Nestin^lo^, CD319^lo^, CD117^hi^, and CD338^lo^) and PC5 (Bcl-6^hi^, Sox2^hi^, KLF-4^hi^, CD289^lo^, Blimp-1^hi^, sXBP-1^hi^, FGFR3^lo^, and Nanog^lo^). Immunophenotypic analysis based on the cell surface markers identified PC6 (very high CD138) and PC7 (IgD^+^) clusters, which differ from other PC clusters but share a common signaling signature with upregulation of c-Myc, CD28, Bcl-6 (higher in PC6), Sox2 (higher in PC7), KLF-4, and CD243 compared with HDs. In addition, upregulation of IRF-4, CD289, Nestin, Blimp-1, CD24, MYD88, sXBP-1, FGFR3, and Nanog associated with downregulation of CD44, CD362, and CD329 was unique in PC6 cluster compared with PC7 cluster (Figure 6, A and B; and Supplemental Figure 6). Comparing the signaling markers either upregulated (red circles in the table) or downregulated (blue circles in the table) among MM stages (Figure 6C), we found 4 markers (CD200, sXBP-1, MMSET, and Bcl-6) overexpressed only in SMM and 7 markers (IRF-4, Nestin, CD319, Sox2, RARa2, CD289, and c-Myc) modulated in NDMM, whereas the modulation of 5 molecules (CD28, MMSET, Nanog, Nestin, and CD289) was noted among MGUS, SMM, and NDMM in different PC clusters (Figure 6C). The variable expression of signaling markers (*n* = 19) even from the MGUS stage is consistent with clonal heterogeneity already present in premalignant stages.

### Correlation of aberrant signaling markers with clinical outcome.

**Figure 7:**
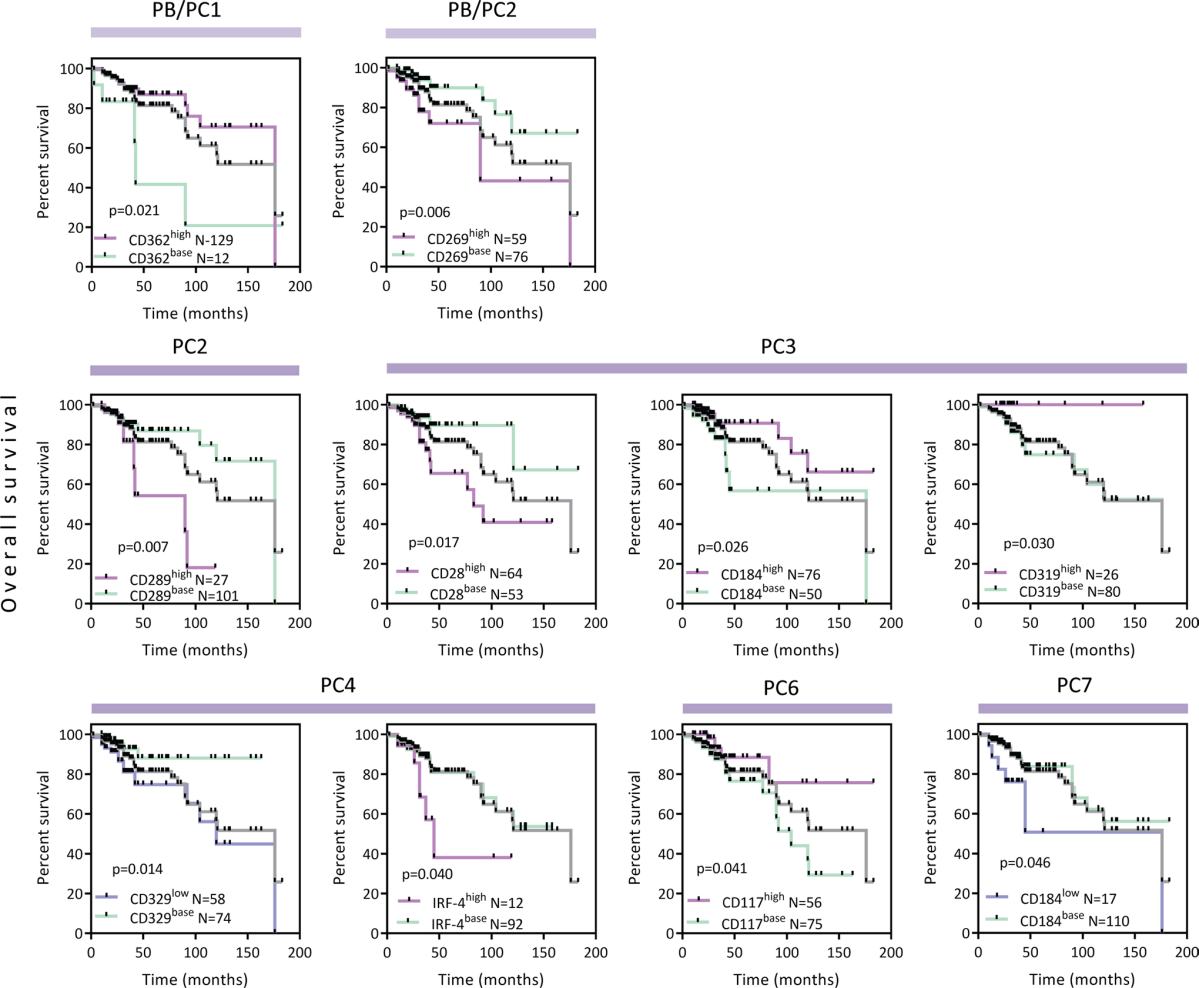
The OS of patients with MM correlated with aberrant expression level of signaling markers. Kaplan-Meier analyses of patients with MM OS according to aberrant expression level of signaling markers (CD362, CD269, CD289, CD28, CD184, CD319, CD329, IRF-4, and CD117) compared with HDs. Red curve represents higher expression than HDs and purple curve is lower expression than HDs; the expression similar to HDs is green curve. There were statistically significant differences in survival correlated in PB/PC clusters (PB/PC1–2) and PC clusters (PC2–4, 6–7), calculated by Kaplan-Meier log-rank test (*P value < 0.05, *n* = 169, gray line).

To delineate potential clinical relevance of profiling studies, we next determined whether signaling in each cluster of PBs and PCs of individual patients correlated with progression-free survival (PFS) and overall survival (OS). Specifically, high levels of Bcl-6 (PB/PC2), CD200 (PC1), sXBP-1 (PB/PC2), IRF-4 (PB/PC2 and PC1–2, 4), c-Myc (PC2), KLF-4 (PC2), Nanog (PB/PC2 and PC2), FGFR3 (PC3), Blimp-1, CD243 (PC4), CD81 (PC5), and CD329 (PC7), as well as lower levels of Sox2 (PB/PC1), FGFR3 (PC2), and CXCR4 (PC5, 7), were associated with shorter PFS. In contrast, higher levels of CD52 (PB/PC1), CXCR4 (PB/PC2), and Bcl-2 (PC7), as well as low levels of CD44 (PC1), CD319 (PC2), CD81 (PC3), and KLF-4 (PC4), were associated with longer PFS (Supplemental Figure 7). High levels of CD362 (PB/PC1) in PBs were associated with better patient OS, while high CD269 (PB/PC2) was significantly associated with an increased risk of death. In PC clusters, high levels of CXCR4, CD319 (PC3), and CD117 (PC6) were associated with longer OS, in contrast to high levels of CD289 (PC2), CD28 (PC3), and IRF-4 and low levels of CD329 (PC4) and CXCR4 (PC7) related to short OS (Figure 7). These data suggest the potential clinical relevance of our in-depth characterization.

## Discussion

Ab-producing PCs are critical effectors of the adaptive immune system and early-stage PCs (PBs) migrate from the tissues of origin to the BM where they either rapidly undergo apoptosis after a few days of intensive Ab secretion (short-lived PCs) or reside in specialized niches, where they evolve to long-lived PCs that survive for many years (12). Immunophenotypic expression profile of PBs is characterized by CD20^–^CD19^+^CD38^+^CD45^+^CD138^+/–^, whereas normal PCs lack pan B cell markers (CD20 and CD22) and mIg and show heterogeneous expression of CD19, CD27, CD45, and polyclonal cytoplasmatic light chains (κ, λ). In contrast, malignant PC typically show underexpression of CD19, CD27, CD45, and CD81; overexpression of CD28, CD33, CD56, CD117, and CD200; and asynchronous expression of CD20 and mIg (13). In our premalignant and active MM cohorts, PBs (CD20^–^CD27^het^CD38^+^CD45^+^CD138^het^) exhibited upregulation of B cell memory CD27 and mIg expression. Similarly, tumor PCs (CD19^het^CD20^het^CD27^het^CD38^+^CD45^lo^CD138^+^) preferentially showed an increase in expression of B cell marker CD20 and mIg (IgA, IgG, and IgM) in premalignant stages, with downregulation of several markers including CD19, CD27, CD38, and CD45 in both NDMM and RRMM. The highest number (*n* = 7) of subsets defined by immunophenotypic differences was determined in PCs compared with 2 subsets of PBs. The highest incidence of PC2 subset, which co-associated with PC1, PC3, PC4, and PC6 clusters, dominated in SMM, NDMM, and RRMM stages.

In-depth profiling of intra- and interneoplastic heterogeneity of malignant PCs of patients versus HDs revealed constitutively overexpressed MMSET, CD47, and Notch-1 across all PB and PC subsets. MMSET (WHSC1) is a member of the nuclear receptor-binding SET domain histone methyltransferase family and as an oncogene regulates many cellular (cell death, cell cycle, and DNA repair) and molecular (p53 pathway, c-Myc, SLAMF7/CS1, NF-κB, Sall1, Sall4, and Nanog) processes (14, 15). Moreover, MMSET, which is identified by fusion to the IgH locus in patients with MM with t(4;14) translocation, is associated with a very poor prognosis (16). The transformation from MGUS to MM is associated with overexpression of Notch-1 (17); however, our data have shown upregulation of Notch-1 in PBs and PCs even in MGUS. Activation of Notch-1, mediated either by MM cells or Jagged ligands produced by stromal cells or stromal mediated cytokine release, stimulates MM proliferation, inhibition of apoptosis, and drug resistance (18). Another potential target we have shown is “don’t eat me” molecule CD47 overexpressed in myeloma B cell lymphopoiesis, similar to diffuse large B cell lymphoma where its blockage increases the phagocytic activity of tumor-associated macrophages expressing inhibitory receptor signal-regulatory protein α (19). Targeting CD47 is under evaluation to eradicate myeloma-initiating cells (20). Interestingly, CD47 expression was increased on PBs, but not on PCs (except PC6 subset), in MGUS, while its upregulation on PCs in SMM, NDMM, and RRMM suggests its importance in the transformation from MGUS to MM and progression within MM.

Several transcription factors are thought to control PB and PC development (12). Plasmacytic differentiation is initiated by activation of the transcription factor IRF-4 (21). Importantly, a hallmark of MM genesis, IRF-4 has been upregulated in PBs of our premalignant and active MM stages. Similar to previously published data (22, 23), IRF-4 increased in the most dominant PC2 subset at NDMM stage, often as a result of activating mutations or translocations. Moreover, high levels of IRF-4 in several subsets of PBs and PCs were associated with shorter OS and PFS. In normal plasmacytic differentiation, IRF-4 causes upregulation of a transcriptional repressor Blimp-1, which is required for the development of Ig-secreting cells and maintenance of long-lived PCs and downregulation of Bcl-6, thereby promoting B cell development in germinal centers by blocking plasmacytic differentiation and sustaining cell cycle progression (24). In our premalignant and active MM stages, increase in Bcl-6 expression was observed in PC subsets, whereas only minimal Blimp-1 expression was observed in RRMM. Once PC differentiation is turned on, Blimp-1 enhances IRF-4 expression and represses Myc (v-myc myelocytomatosis viral oncogene homolog, c-Myc) transcription, causing an arrest in the PC cell cycle (21). Moreover, IRF-4 transactivates the expression of Myc (which encodes the proto-oncoprotein c-Myc) in MM and vice versa, thereby forming a regulatory loop that enforces high IRF-4 (22, 23). In addition, MYC activation is one of the central molecular events leading to MM progression, which is exhibited through various mechanisms (9, 25, 26). We also showed overexpressed c-Myc in PBs and several subsets of PCs across our premalignant and active MM stages. During PC differentiation, Blimp-1 also inhibits the expression of master regulator of B cell identity protein Pax-5, which regulates IRF-4, BTB domain and CNC homolog 2, and activation-induced cytidine deaminase expression during B cell development (27). Consequently, Pax-5 activates expression of XBP-1, a transcription factor required for PC development that induces unfolded protein response target genes associated with high Ig expression in PCs (28). XBP-1 is frequently overexpressed in MM, and high ratios of spliced versus unspliced XBP-1 mRNA directly correlate with lower median OS of patients with MM (29). Our data revealed a potentially unique signature of some PB and PC subsets defined by upregulation of sXBP-1 in SMM. In addition, high aberrant levels of signaling regulators Bcl-6, sXBP-1, c-Myc, and Blimp-1 in some subsets of PBs and PCs were related to inferior PFS.

CXCR4 (CD184) is highly expressed on several B cell subsets, with the highest amounts of CXCR4 on germinal center B cells and decreased expression on normal PBs and PCs (30). Due to the involvement of CXCR4 in normal PC development (31), it has an important role in the expansion and colonization of malignant PCs in the BM (32). However, persistent chemoresistant PC clones (33) and quiescent MM stem cells (34) express high levels of CXCR4, implicated in both disease progression and emergence of evolved subclones. Our data revealed that CXCR4 was upregulated on almost all PC subsets and PBs in our MM cohort. High levels of CXCR4 on PBs and PC subsets were associated with a prolonged OS and PFS, while low levels of CXCR4 on PC subsets were related to an inferior PFS and OS outcome. In MM, MYD88 serves as an adapter protein for TLRs and an activator of NF-κB signaling that is not due to MYD88 mutation, as in diffuse large B cell lymphoma and Waldenström’s macroglobulinemia (35, 36); in our study, we observed its upregulation only in PBs of both premalignant and active MM stages. Constitutively elevated levels of P-glycoprotein (CD243), ABC transporter, were detected in most PBs and PC subsets (related to a worse PFS in PC4), including all stages of MM B cell lymphopoiesis across all MM cohorts; however, no change or even a slight downregulation of breast cancer resistance protein (CD338) has been observed (37).

Several markers with downregulated expression on normal PBs and PCs have aberrant expression on MM cells. Among them are immune checkpoint molecule CD200 (known as the OX-2 tumor antigen) and T cell costimulatory receptor CD28, which correlates with an inferior outcome in patients with MM (38–40). In contrast, aberrant expression of adhesion molecule CD56 and CD117 (proto-oncogene c-kit; its high levels associated with a better OS in our patients with MM), related to an enhanced anchor through kit ligand expression on stromal cells in BM niches, is associated with a favorable outcome (41). In our study, most PC subsets overexpressed CD200 (related to a poor PFS in PC1) and CD28 (related to a worse OS in PC3) in both premalignant MGUS/SMM and active MM, while fewer PC subsets showed an increase in CD117 (both premalignant and active MM stages) and CD56 (active MM) expression. Our data demonstrated that the highly expressed lymphocytic activation molecule family member 7 (CD319, also known as SLAMF7/CS1) on PCs was considerably more robust than CD138; enabled identification of myeloma PCs, including in samples that have been delayed or frozen (42); and was an effective target for new immunotherapies, including CAR T cells and monoclonal Abs (e.g., elotuzumab) (39, 43, 44). We identified increased expression of CD319 in PBs and PC2 subsets, whereas a slight downregulation in the PC4 subset was observed. Another important receptor, CD44, which is expressed on normal PCs and mediates the interaction of PCs with the extracellular matrix molecule hyaluronan, is downregulated as a standard CD44 isoform on malignant PCs (45). Our data showed upregulated CD44 and CD81 expression on normal PBs/PCs in a similar manner and downregulation of both CD81 and CD44 on malignant PBs/PCs in our MM cohorts. Decreased CD44 expression was associated with prolonged PFS in our patients with MM. As in prior studies (46), high CD81 expression on tumor PCs was associated with inferior PFS in our patients with MM. In addition, upregulation (on PB/PC2 and PC6) and downregulation (on PC4/5 subsets) of CD289 (TLR9) (47) expression and CD269 (B cell maturation antigen, also known as BCMA or TNFRSF17) (48) (on PC2 of SMM and active MM stages) were noted. Furthermore, CD362 (SDC2, syndecan proteoglycan family) and CD329 (Siglec-9, Siglec family) are highly expressed on normal PCs (30), but we observed decreased expression on malignant PBs and PCs. Therefore, our study of aberrant PC markers shows that high expression of CD319, CD117, and CD362 was associated with a superior OS, while high levels of CD269, CD289, and CD28 and low levels of CD329 in some PBs and PC subsets were associated with inferior OS.

Fully differentiated somatic cells can be reprogrammed into inducible pluripotent stem cells by “forced” expression of pluripotency/reprogramming factors, Oct3/4, Sox2, Myc, and Klf4, while Nanog is dispensable (49, 50). Reprogramming of mature B cells requires additional “sensitization” by myeloid transcription factor C/EBPα, which causes a specific knockdown of Pax5 or disrupts its functions to promote the gain of stem cell features for mature PCs (51). Moreover, Sox2, Oct3/4, and Nanog are essential for the maintenance of cancer stem cell–like side population cells in MM (52, 53). Importantly, Sox2 is essential for clonogenic growth of CD138^lo^ stem-like cells; anti-Sox2 T cell immunity in MGUS patients efficiently inhibits the clonogenic growth of MGUS cells (54, 55). Expression of the neural stem cell marker Nestin and RARα2 is increased in MM disease progression, especially RARα2 in MM stem cells (56); however, we observed downregulation of RARα2 expression in PCs from active MM stages. KLF-4 is a transcription factor with anti-proliferative effects in differentiated cells; in PC malignancies it acts as a tumor suppressor by upregulating p21^CIP^ and downregulating c-Myc and cyclin D2 and is also associated with melphalan and carfilzomib drug resistance (57). In our study, we defined a potentially unique signature of stemness-controlling markers such as Nestin, Sox2, KLF-4, and Nanog, either up- or downregulated, in PC subsets. This heterogeneity was also correlated with PFS: high levels of KLF-4 (PC2), high (PC3) and low (PC2) levels of FGFR3, high levels of Nanog (PB/PC2 and PC2), and low levels of Sox2 (PB/PC1) were associated with poor clinical outcome, while low levels of KLF-4 (PC4) in PC subsets were related to superior PFS. Overall, our results, therefore, suggest that different PC clusters (or PC subsets) with variations in phenotypes, signaling, and abundance reflect the clonal/subclonal heterogeneity in MM.

The deregulated B cell differentiation under influence by the microenvironment may be key in the emergence of myeloma and its premalignant stages. Plasticity is driven by malignant PCs that share properties of different maturation steps, including B cell precursors. By phenotypic dissection of myeloma B cell lymphopoiesis, we identified a significant increase in expression of CD34 and CD38 on early B cell progenies (hsc, pre-pro-B, and pro-B) and of CD19 on pre-B cells (both pre-BI and pre-BII) in both premalignant and active MM stages, whereas decreased CD10 (known as common acute lymphoblastic leukemia antigen, CALLA) expression was observed only on pre-BII cells of premalignant MM stages, indicating immunophenotypic aberrations already at the first step of B cell precursor differentiation. In B cell maturation, immature B cells first express a complete IgM molecule that undergoes VDJ rearrangement to generate functional B cell receptor precursors (58). In our MM cohort, immature B cells showed decreased expression of IgM. In the transition of immature to mature B cells, fewer changes were detected, except in the RRMM stage, which could reflect the impact of therapies. Mature naive B cells coexpressed IgM and IgD, with CD23 marker associated with transition from immature B cells to naive (mature) B cells. We detected increased CD23 expression ranging from immature B cells to unswitched memory B cells in all MM stages. In the secondary lymphoid organs, mature B cells undergo several processes (such as affinity maturation, somatic hypermutation, and class-switch recombination) that result in the production of long-lived memory B cells or PBs (58). Memory B cells are either unswitched that still coexpress IgM and IgD (sIgM^+^IgD^+^), or potentially IgM (sIgM^+^) only, or switched IgG, IgA, or even IgE memory B cells (5, 59). We identified significant upregulation of memory B cell marker CD27 expression on both unswitched (associated with modulation of CD19 and CD22 in SMM and both active MM stages and overexpression of IgD and IgM in MGUS) and switched memory B cells (along with mIg, such as IgM, IgG, and IgA in the MM cohort), supporting immunophenotypic changes in memory B cells of MM. We also identified signaling modulations in myeloma B lymphopoiesis from hscs to switched memory B cells to PBs or PCs; significantly elevated levels of MMSET, MYD88, c-Myc, CD243, Notch-1, and CD47 were noted even in premalignant conditions, with variable IRF-4 levels within MM stages and B cell subsets. We observed upregulated expression of CD52 from hscs up to unswitched memory B cells in all MM cohorts, whereas downregulated expression of CD44 in translational B cells was demonstrated in active MM stages. Moreover, expression of prosurvival factor Bcl-2 and aberrant PC markers (CD200, CD81, and CD269) in hscs was markedly increased in the MM cohort, reflecting clonal hematopoiesis. A similar signaling profile was observed between hscs and early B cell progenies (early/late pro-B) associated with upregulation of FGFR3, Bcl-6, CD25 (IL-2 receptor α chain, type I transmembrane protein), adhesion molecule CD117, and sXBP-1, along with downregulation of RARα2 and CD362. Furthermore, CXCR4 expression was upregulated from pro-B cells to PCs across all MM cohorts. Bcl-6 was also upregulated in myeloma-switched memory B cells. Moreover, upregulation of activation molecule CD81 was observed in B cell subsets, whereas decreased CD81 expression was detected on switched memory B cells in active MM stages as well as on PBs and PCs in both premalignant and active MM conditions. Elevated levels of stem cell markers were observed in some myeloma B cell subsets, namely Nanog (in hscs and pre-pro-B cells, immature B cells, and unswitched and switched memory B cells), KLF-4 (in hscs and pre-pro-B cells, and from immature B cells to switched memory B cells), Oct3/4 (in B cell precursors pre-pro-B and pre-BI/II cells), and Sox2 (in naive B cells and switched memory B cells). Moreover, we observed a complex pattern of aberrant PC marker upregulation in some subsets of B cells, such as CD200 (pre-BI, transitional, and naive B cells), CD28 (naive B cells and switched memory B cells), sXBP-1 (in the B cell precursors pre-pro-B and pro-B cells, transitional B cells, and unswitched memory B cells), and CD319 (switched memory B cells) in our MM cohort. Collectively, these data revealed phenotypic and signaling variations within premalignant and active myeloma B cell lymphopoiesis, corresponding with clonal hematopoiesis.

Identification of the critical network of B cell regulatory signaling, stemness-controlling markers, and other aberrant activators of B cell lymphopoiesis that surround malignant transformation and progression may provide important insight into myeloma disease. In addition, defining cellular and molecular subclonal heterogeneity of PCs, in both premalignant and active stages, represents a major goal to enhance understanding of the pathogenesis of MM. This study provides a framework for delineation of network signaling characteristics and their associated mechanistic or clinical outcomes.

## Methods

### Experimental model and patient details.

BM aspirates of a total of 188 patients with MM (premalignant/asymptomatic MGUS [*n* = 16] and SMM [*n* = 25]; and active symptomatic stages NDMM [*n* = 43] and RRMM [*n* = 104]) and HDs (*n* = 10) were enrolled in this study. The clinical characteristics of patients with MM are listed in Supplemental Table 1. In addition, all clinical and medical information of patients with MM was gathered at the time of collection, including diagnosis and the history of treatment. BM samples (*n* = 188) were collected from patients at the Dana-Farber Cancer Institute and Biomedical Research Center who were undergoing routine diagnostic BM aspiration. Healthy BM control samples from age-matched donors (*n* = 10) were obtained from AllCells using the same collection protocol.

### Statistics.

The tests of normality and the Kolmogorov-Smirnov and Shapiro-Wilk tests were used to assess distribution of data. The outliers were identified by Tukey’s test. Statistical significance of 2 groups was determined by the nonparametric Mann-Whitney *U* test. The differences in median values among 4 MM stages versus the HD control groups were evaluated by Dunn’s multiple comparison test after the Kruskal-Wallis 1-way ANOVA by ranks, with *P* value < 0.05 considered significant.

### Study approval.

The study was approved by the local ethics committee of the Biomedical Research Center in Bratislava, Slovakia (protocol number Myelom 001 for MM cohort) and the Dana-Farber Cancer Institute in Boston, Massachusetts, USA (protocol number 10-106 for MM cohort) in accordance with the Declaration of Helsinki protocol. All patient and healthy donor samples were taken after receipt of written informed consent to donate a portion of the sample for this study in accordance with the Declaration of Helsinki protocol.

Supplemental Methods, including all experimental procedures and CyTOF data analysis, are available online with this article.

## Author contributions

JJ conceived and designed the study, performed experiments, analyzed data, and wrote the manuscript. DC performed experiments, analyzed data, and contributed to the writing of the manuscript. GB and LK performed bioinformatics analyses and contributed to the writing of the manuscript. TH contributed to the study design. ML, KJ, PGR, EK, and DMD contributed with clinical specimens and collected clinical data. KCA wrote and edited the manuscript.

## Supplementary Material

Supplemental data

## Version 1 02/08/2023

Electronic publication
