# Supplementary material for: Heterogeneity of B cell lymphopoiesis in patients with premalignant and active myeloma
Source: JCI Insight. 2023 Feb 8;8(3):e159924. doi: 10.1172/jci.insight.159924 (PMC9977435; doi:10.1172/jci.insight.159924)
Supplement: Supplemental data [file jciinsight-8-159924-s105.pdf]

# SUPPLEMENTARY INFORMATION

## Heterogeneity of B cell lymphopoiesis in premalignant and active myeloma patients

Jana Jakubikova, Danka Cholujova, Gabor Beke, Teru Hideshima, Lubos Klucar, Merav Leiba, Krzysztof Jamroziak, Paul G Richardson, Efstathios Kastritis, David M Dorfman and Kenneth C Anderson

**Table of Contents:**

| | |
|---|---|
| Supplementary Methods | 1 |
| References | 11 |
| Supplementary Table 1 | 12 |
| Supplementary Table 2 | 13 |
| Supplementary Table 3 | 14 |
| Supplementary Figure S1 | 15 |
| Supplementary Figure S2 | 16 |
| Supplementary Figure S3 | 18 |
| Supplementary Figure S4 | 19 |
| Supplementary Figure S5 | 20 |
| Supplementary Figure S6 | 21 |
| Supplementary Figure S7 | 22 |

## SUPPLEMENTARY METHODS

### Human healthy peripheral blood mononuclear cells and cell lines

Multiple myeloma cell lines RPMI 8226-S (also referred to as RPMI-S) and MM.1S were obtained from ATCC (Manassas, VA). The RPMI 8226-S sublines resistant to doxorubicin (RPMI-Dox40) and mitoxantrone (RPMI-MR20) were kindly provided by Dr. Teru Hideshima (Dana Farber Cancer Institute, Boston, MA, USA). Similarly, RAJI (human Burkitt′s lymphoma), KG-1a (human acute myelogenous leukemia), Jurkat (human acute T cell leukemia), MOLT-4 (human acute T lymphoblastic leukemia), HeLa (human cervical epithelial adenocarcinoma) and NTERA-2 (human pluripotent embryonic carcinoma) were obtained from ATCC (Manassas, VA). All cell lines were cultured in Roswell Park Memorial Institute medium (RPMI 1640) medium (Cellgro, Mediatech, VA), except for NTERA-2 cultured in Dulbecco's modified Eagle medium (DMEM; Cellgro, Mediatech, VA), supplemented with 10% heat-inactivated fetal bovine serum (FBS; Harlan, Indianapolis, IN), 100 U/mL penicillin, 100 μg/mL streptomycin, and 2 mM L-glutamine (GIBCO, Grand Island, NY) at 37 °C in 5% $CO_2$. Fresh peripheral blood mononuclear cells (PBMNCs) were obtained from healthy volunteers by Ficoll-Hypaque (Pharmacia, Piscataway, NJ) density sedimentation. Mononuclear cells were cultured in RPMI 1640 containing 20% heat-inactivated FBS, 100 U/mL penicillin, 100 μg/mL streptomycin, and 2 mM L-glutamine, and then maintained at 37 °C in 5% CO2. Informed consent was also obtained from all healthy volunteers, in accordance with the Declaration of Helsinki protocol. Granulocyte-macrophage colony-stimulating factor (GM-CSF; at 50 ng/ml concentration for 7 days) and campthotecin (at 6 μM concentration for 8 hours) were used for stimulation of PBMNCs and MM.1S, respectively.

## EXPERIMENTAL PROCEDURES

### Antibodies and antibody conjugation

Antibodies with clones, providers and concentrations are listed in Supplementary Table S2. Antibodies (100 µg antibody lots) were conjugated to the MaxPAR antibody conjugation kit with respective mass isotopes (Fluidigm Sciences, San Francisco, CA) as shown in Supplementary Table S2, according to the manufacturer's instructions. Briefly, the polymer was loaded with lanthanide metal solution at concentration 2.5 mM for 40 min at 37 °C. During the incubation, the antibody (100 µg) buffer was exchange and a partial reduction of the antibody was performed with 100 µl of 4 mM bond-breaker TCEP (tris(2-carboxyethyl)phosphine) for 30 min at 37 °C. After two purification steps of both lanthanide-loaded polymer and partially reduced antibody, the antibody was conjugated to lanthanide-loaded polymer for 1 hour at 37 °C. Then, following 4 steps of washing the metal conjugated antibody was recovered.

### Evaluation of metal-conjugated antibody efficacy

To evaluate metal-conjugated antibody efficacy, metal-conjugated antibody was digested at dilution 100-fold in 2% hydrochloric acid (HCl, Seastar Chemicals, British Columbia, Canada) diluted in double-distilled water (ddH$_2$O), followed with 100-fold dilution in 2% nitric acid (HNO3, Seastar Chemicals, British Columbia, Canada) diluted in ddH$_2$O. The metal content of labeled antibodies (with ≥ 100 metal atoms per antibody) was determined with CyTOF 2 mass

cytometer (Fluidigm, San Francisco, CA). Diluted metal-conjugated antibodies were acquired on the CyTOF 2 in solution mode and the metal content of the conjugated antibody was calculated against blank and tuning solution (Fluidigm Sciences, San Francisco, CA) used as the normalization standard in calculations. In addition, the quantity of each antibody was assessed after metal conjugation at 280 nm absorbance using a NanoDrop (Thermo Scientific, Wilmington, Delaware). After concentration assessment, antibodies were diluted to a final concentration of 0.5 mg/mL in Candor PBS antibody stabilization solution (Candor Bioscience GmbH, Wangen, DE) supplemented with 0.05% sodium azide ($NaN_3$) and stored at 4 °C. All antibody-metal conjugates were titrated for optimal concentration of use and evaluated for their efficacy using positive controls, either human peripheral blood mononuclear cells or human cell lines, listed in Supplementary Table S2.

**Sample collection**

Fresh BM aspirates were collected immediately (< 1 minute) after aspiration into sodium heparinized tubes (BD Biosciences, San Jose, CA). Samples were then fixed with proteomic stabilizer buffer (Smart Tube, San Carlos, CA), according to the manufacturer's instructions by adding 1.4 ml of proteomic stabilizer buffer to 1 ml of BM sample and incubating for 10 min at room temperature (RT) on a rotator, and then frozen at –80 °C. Bone marrow samples were thawed just prior to analysis in a 4 °C cold water bath. Erythrocytes were lysed by hypotonic lysis "1 x thaw-lyse" buffer (1000-fold diluted concentrate with distilled water; Smart Tube, San Carlos, CA) in a ratio 4:1 to the total volume of the sample and incubated for 10 min at RT. To pellet leukocyte samples were centrifuged at 600 x g for 6 min at RT and the supernatant was discarded.

For complete lysis of erythrocytes, steps with 1x thaw-lyse buffer and its removal were repeated. Cells were then washed twice in cell staining medium (CSM; 1 × phosphate buffered saline (PBS) with 0.5% bovine serum albumin (BSA) and 0.02% $NaN_3$) and collected at 600 x g for 6 min at RT.

**Cell sorting**

Samples were thawed as previously described and a part of sample aliquot was stained with anti-human CD15-PerCP-Cy5.5 mouse antibody (clone HI98; BD Pharmingen, San Jose, CA, USA) for 30 min at 4 °C. Cells were sorted for CD15 negative cells to deplete a high percentage of granulocyte CD15+ cells using a 70-µm nozzle on a FACS Aria III 5L cell sorter. Cells were harvested in CSM and used immediately for CyTOF sample preparation.

**CyTOF sample preparation**

Cells were washed in cell staining media (CSM; PBS with 0.5% BSA and 0.02% $NaN_3$) and collected by centrifugation at 600 x g for 6 min at RT. At this point, samples were split into three sample aliquots for concurrent cell staining with three antibody panels (5 x $10^6$ cells in 50 µl CSM per panel). Before staining with cell surface marker antibody cocktails of respective panel, cells were incubated with 5 µl of Fc-receptor blocking solution (human TruStain FcX; BioLegend, San Diego, CA) for 10 minutes at RT to block non-specific binding, and then labeled with cell surface antibody cocktail to a final volume of 100 µl by CSM for 30 min at RT. An aliquot of the sample

was stained with BI panel of cell surface antibodies, the other aliquot sample was stained with BII panel of cell surface antibody cocktail (either sorted or unsorted). After staining, cells were washed twice in CSM and centrifuged at 600 x g for 6 min at RT. For intracellular staining, samples were either permeabilized with 1 mL of 4 °C cold methanol and stored at -80 °C. Then, cells were washed twice in CSM to remove the remaining permeabilization solution, and stained with intracellular antibody cocktails according to the appropriate panel (BI or BII panel) and 1 μL of 191/193 Iridium (Ir) DNA intercalator (Fluidigm, San Francisco, CA) in a final volume of 100 μl by CSM for 1 hour at RT. Cells were then washed twice in CSM and collected by centrifugation at 600 x g for 6 min at RT.

**CyTOF data acquisition**

Immediately prior to acquisition, cells were washed with PBS alone and then with dd$H_2O$. Cells were diluted to 0.5 x $10^6$ cells/ml in dd$H_2O$ containing 10% of EQ Four Element Calibration Beads (Fluidigm, San Francisco, CA). Samples were acquired on a CyTOF 2 mass cytometer (Fluidigm, San Francisco, CA) at an even rate of 300-500 events per second with instrument-calibrated dual-count detection.

## QUANTIFICATION AND STATISTICAL ANALYSIS

### CyTOF data analysis

Individual .fcs files collected from each set of samples were concatenated using the .fcs concatenation tool from Cytobank (1) (Mountain View, CA), and data were normalized using the Normalizer tool with EQ Four Element Calibration Beads (2) to correct for signal fluctuations within each experiment and across all batch analyses (Supplementary Figure S1A). Signal intensities for each channel were arcsinh transformed with a cofactor of 5 (x_transf =asinh(x/5)). All gating strategies and extraction of median expression level were defined using Cytobank software (Mountain View, CA). First, the populations of interest were manually gated based on the visualization of biaxial marker expression by hierarchy of manual gating (Supplementary Figure S1B). Using biaxial 191Ir (DNA1) and 193Ir (DNA2) marker expression cells (to avoid debris) were identified followed by gating on singlets (to remove doubles). Similarly, in following gating strategy calibration beads were removed from the analysis based on gate "singlets w/o beads''. Then, gate on viable cells based on negative expression of cleaved caspase-3 and cleaved PARP, which were used as cell surface marker labeling, was defined. The final manual gate (w/o CD3&CD14&CD15) was defined either by removing the entire CD3+/CD14+/CD15+ population in unsorted samples or CD3+/CD14+ population in sorted samples where CD15+ population was previously sorted out (Supplementary Figure S1B). Furthermore, w/o CD3&CD14&CD15 (in BI and BII panels) gate was used for further high-dimensional clustering analyses either performed by spanning-tree progression analysis of density-normalized events (SPADE).

## The SPADE implementation

High-dimensional data was analyzed by spanning-tree progression analysis of density-normalized events (SPADE), a computational approach that organized cells by a hierarchy of related phenotype into a branched tree structure implemented in Cytobank software (Mountain View, CA) (3). Briefly, the SPADE algorithm consists of 4 computational modules: (i) density-dependent down-sampling to equalize the density in the point cloud of cells (to remove density variation), (ii) agglomerative clustering to partition the point cloud of cells into cell clusters, (iii) minimum spanning tree construction to link cell clusters, and (iv) up-sampling to map all the cells onto the resulting tree structure (4). SPADE organizes cells to the cluster in the tree by a hierarchy of related phenotype. Each node of the SPADE tree is colored according to median intensity of the respective marker, from low expression (blue color) to high expression (red color), allowing visualization of the behavior of the marker across the entire heterogeneous cell population. Moreover, the size of each node is correlated to the fraction of cells mapping to the node. The input parameters of SPADE analysis included (i) 13 cell surface markers as clustering markers in BI & BII panels, (ii) 1% outlier density to exclude outliers, (iii) 10% target density to retain 10% cells after down-sampling process of SPADE analysis, and (iv) number of clusters: 300 in BI & BII panels to define desired number of clusters. In addition, all BM samples of MM patients and healthy donors in BI and BII panels were analyzed simultaneously by SPADE approach, generating the same tree structure, so the resulting tree structure captured all subpopulations present in the entire dataset. Furthermore, SPADE was used to compare multiple samples, with overlapping staining in BI and BII panels. After separately downsampling the data, we can pool the downsampled data into a meta-downsampled dataset, which is a meta-cloud that represents where a cell is in a high-dimensional space defined by the markers that are common to both panels. Therefore, the shape

of the SPADE tree was defined by 13 overlapping clustering markers in both BI and BII panels. For markers that varied across panels, its behavior can be visualized by contrasting its intensities on differently colored samples. The boundaries to separate the clusters that show drastically different colors in SPADE tree were drawn manually and annotated into immunophenotypic populations, according to the colored tree and based upon examination of positive *versus* negative expression of relevant biaxial plots of cellular events in each cluster. Prior knowledge was used to interpret the biological relevance of individual tree clusters.

**CyTOF data visualization**

For interactive visualization of the results, we developed a web portal using R software and its additional packages, mainly *shiny*. Shiny is an R library that allows the development of a simple interactive web application inside R. The application is accessible via any web browser, facilitating server computing resources. The other R packages used for the development include *shinyBS*, *igraph*, *ggplot2*, *Cairo*, *gplots*, *colorRamps*, *plotly* and *DT* (Supplementary Table 3). The main reason behind the development of this web portal was to annotate clusters of different cell populations in SPADE analyses. After annotation of the SPADE trees, we normalized the number of cells in each cell population to the total number of cells in the sample. To normalize the number of cells we used the following formula:

$$x = cB/cA,$$

where x is the normalized number of cells in the cluster of cell population, cB is the sum of the number of cells from all nodes belonging to one cluster, cA is the total number of cells from all nodes in all clusters.

We also normalized the median expression for each marker in each sample. To normalize the expression we used the formula:

$$y = \Sigma\,(c1.\,e1 + c2.\,e2 + \cdots + cn.\,en)\,n1/cB,$$

where y is the normalized median expression of one marker in one cluster of one sample. c is the number of cells in one node, e is the median expression for one marker in one node and cB is the sum of the number of cells from nodes belonging to one cluster.

For further analysis, we modified the *mergeClusters*, *identifyDAC* and *volcanoViewer* functions from the *SPADEVizR* package ((5) to analyze the marker level among the clusters originally present in this package. We developed another web portal to visualize normalized SPADE results and examine them in different approaches, such as in the form of SPADE trees, heatmaps, dot-plots and whisker/box graphs using R shiny and other packages: *shinydashboard*, *htmlwidgets*, *openxlsx*, *queryBuilder*, *rhtmlHeatmap*, *RColorBrewer*, *viridisLite*, *grid*, *gridExtra* in addition to those listed above. Based on the above-mentioned visualizations, various factors and data, e.g. biological and/or clinical data, we generated different groups of data. These data sets were statistically compared using Mann-Whitney-U test and visualized on volcano plots (*identifyDAC* and *volcanoViewer* functions from the *SPADEVizR* R libary and their modified versions) on this web portal. Both web portals were developed for in-house use only and were run on our Linux-based web server.

To effectively visualize the comparison of the up- or down-regulation of statistically significant markers in multiple cell clusters, we used circular plots (R software and R libraries: *circlize* (6) and *ComplexHeatmap* (7)). Venn diagrams were designed to show common changes

of marker expression in cell cluster at various stages of MM using *VennDiagram* R package. Principal component analysis (PCA), correspondence analysis (CA) and their visualizations were generated in R software and *FactoMineR*, *factoextra*, *survminer* and *survival* R libraries. Correlation heatmaps were created using the *ggcorrplot* R package (Supplementary Table 3).

**Statistical analysis**

The tests of normality, the Kolmogorov-Smirnov and the Shapiro-Wilk tests were used to assess distribution of data. The outliers were identified by Tukey test. Statistical significance of two groups was determined by non-parametric Mann-Whitney U test. The differences in median values among four MM stages versus HD control group were evaluated by Dunn's multiple comparison test after the Kruskal-Wallis one-way analysis of variance by ranks test, with p value $< 0.05$.

**REFERENCES**

1. Chen TJ, and Kotecha N. Cytobank: providing an analytics platform for community cytometry data analysis and collaboration. CurrTopMicrobiolImmunol. 2014;377:127-57.

2. Finck R, et al. Normalization of mass cytometry data with bead standards. Cytometry A. 2013;83(5):483-94.

3. Kotecha N, et al. Web-based analysis and publication of flow cytometry experiments. CurrProtocCytom. 2010;Chapter 10:Unit10.

4. Qiu P, et al. Extracting a cellular hierarchy from high-dimensional cytometry data with SPADE. NatBiotechnol. 2011;29(10):886-91.

5. Gautreau G, et al. SPADEVizR: an R package for visualization, analysis and integration of SPADE results. Bioinformatics. 2017;33(5):779-81.

6. Gu Z, et al. circlize Implements and enhances circular visualization in R. Bioinformatics. 2014;30(19):2811-2.

7. Gu Z, et al. Complex heatmaps reveal patterns and correlations in multidimensional genomic data. Bioinformatics. 2016;32(18):2847-9.

| CHARACTERISTICS | MM PATIENTS (N=188) |
|---|---|
| ***Median age (range) - yr*** | 65 (29-91) |
| ***Sex - no. (%)*** | |
| Female | 83 (44) |
| Male | 122 (65) |
| ***Stage of myeloma - no. (%)*** | |
| ***MGUS*** | 16 (8.5) |
| ***SMM*** | 25 (13) |
| ***NDMM*** | 43 (23) |
| ***RRMM*** | 104 (55) |
| ***Medium serum creatinine (range) - mg/dl*** | 0.955 (0.42-3.33) |
| ***Medium calcium (range) - mg/dl*** | 9.4 (4.3-10.8) |
| ***Medium serum albumin (range) - g/dl*** | 4.2 (1.7-4.9) |
| ***Medium lactate dehydrogenase (range) - U/l*** | 151 (72-550) |
| ***Beta 2 microglobulin (range) - mg/l*** | 2.9 (1.5-18.6) |
| ***Hemoglobin (range) - g/dl*** | 12.3 (7.5-29.9) |
| ***Platelet count (range) - per mm$^3$*** | 199 (37-549) |
| ***M-spike*** | |
| Median (range) - g/dl | 1.15 (0.1-6.33) |
| No. of gamma type (%) | 137 (73) |
| No. of beta type (%) | 12 (6) |
| No. of gamma+beta type (%) | 2 (1) |
| ***Type of myeloma (immunoglobulin)*** | |
| Median IgA (range) - mg/dl | 873 (11-5043) |
| No. of patients (%) | 30 (16) |
| Median IgG (range) - mg/dl | 1524.5 (38-7980) |
| No. of patients (%) | 140 (74) |
| No. of IgD patients (%) | 3 (2) |
| ***Type of light chain*** | |
| Median kappa (range) - mg/l | 46.9 (1.8-7257) |
| No. of patients (%) | 135 (72) |
| Median lambda (range) - mg/l | 89.4 (1.1-6754) |
| No. of patients (%) | 62 (33) |
| ***Median k/l (range)*** | 2.83 (0-5606) |
| ***Median bone marrow involvement (range) - (%)*** | 40 (5-95) |
| ***Previous therapy of RRMM patients*** | |
| Median no. of previous treatment regimens (range) | 0 (0-10) |
| 1 - no. | 33 (32) |
| 2 - 3 - no. | 34 (33) |
| ≥ 4 - no. | 23 (22) |
| ***Type of previous therapy - no. (%)*** | |
| Glucocorticoid | 80 (77) |
| Proteasome inhibitor | 77 (74) |
| Alkylator | 28 (27) |
| Immunomodulator | 96 (92) |
| Antracycline | 6 (6) |
| Vinca alkaloids and derivatives | 6 (6) |
| Other, including experimental therapy | 50 (48) |

Supplementary Table 1: **Clinical characteristics of patients with multiple myeloma at the time of CyTOF analysis.**

| ANTIBODIES | LOCATION | PROVIDER | IDENTIFIER | METALS | PROVIDER | IDENTIFIER | CONCENTRATION USED [mM] per sample | METAL CONTENT DETERMINED [Atoms/Ab] | POSITIVE CONTROL |
|---|---|---|---|---|---|---|---|---|---|
| **13 CLUSTERING OVERLAPING MARKERS IN BI AND BII PANELS** | | | | | | | | | |
| Anti-human CD10 (clone HI10a)-purified | surface | Biolegend | cat#: 312202 | 156Gd | Fluidigm | 201156A | 0.2 | 170.006 | RAJI cells untreated |
| Anti-human CD19 (clone HIB19)-purified | surface | Biolegend | cat#: 302202 | 142Nd | Fluidigm | 201142A | 0.2 | 173.879 | PBMNCs |
| Anti-human CD20 (clone 2H7)-purified | surface | Biolegend | cat#: 302302 | 147Sm | Fluidigm | 201147A | 0.4 | 139.833 | PBMNCs |
| Anti-human CD22 (clone HIB22) -purified | surface | Biolegend | cat#: 302502 | 159Tb | Fluidigm | 201159A | 0.2 | 175.527 | PBMNCs |
| Anti-human CD27 (clone M-T271)-purified | surface | Biolegend | cat#: 356401 | 167Er | Fluidigm | 201167A | 0.8 | 194.630 | PBMNCs |
| Anti-human CD34 (clone 581)-purified | surface | Biolegend | cat#: 343502 | 162Dy | Fluidigm | 201162A | 0.1 | 111.733 | KG-1a cells untreated |
| Anti-human CD38 (clone HIT2)-purified | surface | Biolegend | cat#: 303502 | 152Sm | Fluidigm | 201152A | 0.8 | 178.290 | PBMNCs |
| Anti-human CD45 (clone HI30)-purified | surface | Biolegend | cat#: 304002 | 154Sm | Fluidigm | 201154A | 0.1 | 122.684 | PBMNCs |
| Anti-human CD138 (clone DL-101) -purified | surface | Biolegend | cat#: 352302 | 172Yb | Fluidigm | 201172A | 0.4 | 195.895 | RPMI-S cells untreated |
| Anti-human IgA(clone polyclonal)-148Nd | surface | Fluidigm | 3148007B | | | | 0.1 | | PBMNCs |
| Anti-human IgD (clone IA6-2)-purified | surface | Biolegend | cat#: 348202 | 150Nd | Fluidigm | 201150A | 0.1 | 132.847 | PBMNCs |
| Anti-human IgG (clone G18-145)-purified | surface | BD Pharmingen | cat#: 555784 | 153Eu | Fluidigm | 201153A | 0.2 | 134.957 | PBMNCs |
| Anti-human IgM (clone MHM-88)-purified | surface | Biolegend | cat#: 314502 | 170Er | Fluidigm | 201170A | 0.1 | 174.112 | PBMNCs |
| | | | | | | | | | |
| **ADDITIONAL DUMP MARKERS IN BI AND BII PANELS** | | | | | | | | | |
| Anti-human CD3 (clone UCHT1)-purified | surface | Biolegend | cat#: 300414 | 143Nd | Fluidigm | 201143A | 0.2 | 115.502 | PBMNCs |
| Anti-human CD14 (clone M5E2)-purified | surface | Biolegend | cat#: 301802 | 143Nd | Fluidigm | 201143A | 1.6 | 120.950 | PBMNCs |
| Anti-human CD15 (clone W6D3) -purified | surface | Biolegend | cat#: 323002 | 143Nd | Fluidigm | 201143A | 0.8 | 137.202 | PBMNCs |
| | | | | | | | | | |
| **ADDITIONAL OVERLAPING MARKERS IN ALL PANELS** | | | | | | | | | |
| Anti-human Ig light chain κ (clone MHK-49)- purified | intracellular | Biolegend | cat#: 316502 | 165Ho | Fluidigm | 201165A | 0.05 | 207.662 | PBMNCs |
| Anti-human Ig light chain λ (clone MHL-38) -purified | intracellular | Biolegend | cat#: 316602 | 174Yb | Fluidigm | 201174A | 0.05 | 231.055 | PBMNCs |
| Cleaved PARP (clone D64E10) XP® Rabbit mAb | surface | Cell Signaling Technologies | cat#: 5625 | 113In | | | 0.2 | 99.720 | MM.1S cells treated by campthotecin |
| Cleaved caspase-3 (clone D3E9) Rabbit mAb | surface | Cell Signaling Technologies | cat#: 9579 | 115In | | | 0.4 | 98.458 | MM.1S cells treated by campthotecin |
| | | | | | | | | | |
| **BI PANEL (B CELL DEVELOPMENT PANEL)** | | | | | | | | | |
| Anti-human CD52 (clone HI186)-purified | surface | Biolegend | cat#: 316002 | 139La | Fluidigm | 201139A | 0.4 | 232.799 | PBMNCs |
| Anti-human CD81(TAPA-1) (clone 5A6)-purified | surface | Biolegend | cat#: 349502 | 141Pr | Fluidigm | 201141A | 0.8 | 129.752 | PBMNCs |
| Anti-human CD28 (clone CD28.2)-Ultra-LEAF™ purified | surface | Biolegend | cat#: 302933 | 144Nd | Fluidigm | 201144A | 1.6 | 112.007 | PBMNCs |
| Anti-human CD221 (clone 3B7)-purified | surface | BD Pharmingen | cat#: 556000 | 145Nd | Fluidigm | 201145A | 0.8 | 124.536 | PBMNCs |
| Anti-human CD62L (clone DREG-56)-purified | surface | Biolegend | cat#: 304802 | 146Nd | Fluidigm | 201146A | 0.2 | 104.704 | PBMNCs |
| Anti-human CD117 (clone YB5.B8)-purified | surface | BD Pharmingen | cat#: 555713 | 149Sm | Fluidigm | 201149A | 0.8 | 110.453 | Jurkat cells untreated |
| Anti-TdT (clone L10-2)-purified | intracellular | Biolegend | cat#: 649302 | 151Eu | Fluidigm | 201151A | 0.4 | 140.772 | Molt-4 cells untreated |
| Anti-human CD23 (clone M-L233)-purified | surface | BD Pharmingen | cat#: 555707 | 155Gd | Fluidigm | 201155A | 1.6 | 202.316 | PBMNCs |
| Anti-Blimp-1 (clone ROS195G)-purified | intracellular | Biolegend | cat#: 648202 | 158Gd | Fluidigm | 201158A | 1.6 | 120.208 | RAJI cells untreated |
| Anti-PAX5 (clone D19F8) XP® Rabbit mAb | intracellular | Cell Signaling Technologies | cat#: 8970 | 160Gd | Fluidigm | 201160A | 0.4 | 107.311 | RAJI cells untreated |
| Anti-human CD25 (clone M-A251)-purified | surface | Biolegend | cat#: 356102 | 161Dy | Fluidigm | 201161A | 1.6 | 188.398 | PBMNCs |
| Anti-Bcl-2 (clone BCL/10C4)-purified | intracellular | Biolegend | cat#: 633502 | 163Dy | Fluidigm | 201163A | 1.6 | 205.808 | HeLa cells untreated |
| Anti-FGFR3 (clone MM0279-6G11) | intracellular | Abcam | cat#: ab89660 | 164Dy | Fluidigm | 201164A | 1.6 | 210.593 | HeLa cells untreated |
| Anti-IRF4 (clone IRF4.3E4) | intracellular | Biolegend | cat#: 646402 | 166Er | Fluidigm | 201166A | 1.6 | 196.561 | RAJI cells untreated |
| Anti-human XBP1 (S Isoform) MAb (Clone 525904) | intracellular | R&D Systems | cat#: MAB4257 | 168Er | Fluidigm | 201168A | 0.4 | 107.507 | RAJI cells untreated |
| Anti-WHSC1/NSD2 antibody (clone 29D1) | intracellular | Abcam | cat#: ab75359 | 169Tm | Fluidigm | 201169A | 0.4 | 168.650 | HeLa cells untreated |
| Anti-mouse/human Bcl-6 (clone IG191E/A8)-purified | intracellular | Biolegend | cat#: 648302 | 171Yb | Fluidigm | 201171A | 0.4 | 115.786 | RAJI cells untreated |
| Anti-human MyD88 MAb (clone 316628) | intracellular | R&D Systems | cat#: MAB29281 | 173Yb | Fluidigm | 201173A | 1.6 | 241.203 | RAJI cells untreated |
| Anti-human CD56 (clone HCD56)-purified | surface | Biolegend | cat#: 318302 | 175Lu | Fluidigm | 201175A | 0.4 | 252.482 | PBMNCs |
| Anti-c-Myc (clone D84C12) XP® Rabbit mAb | intracellular | Cell Signaling Technologies | cat#: 5605 | 176Yb | Fluidigm | 201176A | 1.6 | 164.023 | RAJI cells untreated |
| | | | | | | | | | |
| **BII PANEL (MM STEM CELL DISCOVERING)** | | | | | | | | | |
| Anti-human CD44 (clone BJ18)-purified | surface | Biolegend | cat#: 338802 | 139La | Fluidigm | 201139A | 0.2 | 98.654 | PBMNCs |
| Anti-human CD200 (OX2) (clone OX-104)-purified | surface | Biolegend | cat#: 329202 | 141Pr | Fluidigm | 201141A | 0.8 | 169.551 | PBMNCs |
| Anti-human CD47 (clone CC2C6)-purified | surface | Biolegend | cat#: 323102 | 144Nd | Fluidigm | 201144A | 0.2 | 101.610 | PBMNCs |
| Anti-human CD243 (clone UIC2)-purified | surface | Biolegend | cat#: 348602 | 145Nd | Fluidigm | 201145A | 0.2 | 100.728 | RPMI-DOX40 cells untreated |
| Anti-human CD24 (clone ML5)-purified | surface | Biolegend | cat#: 311102 | 146Nd | Fluidigm | 201146A | 0.8 | 134.306 | PBMNCs |
| Anti-human CD184 (CXCR4) (clone 12G5)-purified | surface | Biolegend | cat#: 306502 | 149Sm | Fluidigm | 201149A | 0.1 | 143.413 | HeLa cells untreated |
| Anti-Retinoic Acid Receptor alpha (clone H1920)-ChIP Grade | intracellular | Abcam | cat#: ab41934 | 151Eu | Fluidigm | 201151A | 1.6 | 109.410 | Jurkat cells untreated |
| Anti-human CD362 (syndecan-2) (clone 305515) | surface | R&D Systems | cat#: MAB2965 | 155Gd | Fluidigm | 201155A | 1.6 | 294.013 | PBMNCs |
| Anti-Human CD325 (N-cadherin) (clone 8C11)-purified | surface | BD Pharmingen | cat#: 561553 | 158Gd | Fluidigm | 201158A | 0.8 | 213.238 | HeLa cells untreated |
| Anti-Notch1 (clone mN1A) | intracellular | Abcam | cat#: ab128076 | 160Gd | Fluidigm | 201160A | 1.6 | 165.390 | HeLa cells untreated |
| Anti-human Siglec-9 (CD329) (clone polyclonal) | surface | R&D Systems | cat#:AF1139 | 161Dy | Fluidigm | 201161A | 0.4 | 196.657 | PBMNCs |
| Anti-human CD269 (BCMA) (clone 19F2)-purified | surface | Biolegend | cat#: 357502 | 163Dy | Fluidigm | 201163A | 1.6 | 213.061 | PBMNCs |
| Anti-Nanog (clone D73G4) XP® Rabbit mAb | intracellular | Cell Signaling Technologies | cat#: 4903 | 164Dy | Fluidigm | 201164A | 0.8 | 186.900 | NTERA-2 cells untreated |
| Anti-Sox-2 (clone O30-678)-purified | intracellular | BD Pharmingen | cat#: 561469 | 166Er | Fluidigm | 201166A | 0.2 | 181.828 | NTERA-2 cells untreated |
| Anti-Oct3/4 (clone O50-808)-purified | intracellular | BD Pharmingen | cat#: 561555 | 168Er | Fluidigm | 201168A | 0.1 | 158.168 | NTERA-2 cells untreated |
| Anti-human KLF4 (clone polyclonal)-affinity purified | intracellular | R&D Systems | cat#: AF3640 | 169Tm | Fluidigm | 201169A | 0.8 | 308.796 | HeLa cells untreated |
| Anti-Nestin (clone 2C1.3A11) | intracellular | Abcam | cat#: ab18102 | 171Yb | Fluidigm | 201171A | 0.4 | 196.196 | NTERA-2 cells untreated |
| Anti-human TLR9 (CD289, toll-like receptor, clone polyclonal )-affinity purified | surface | R&D Systems | cat#: AF3658 | 173Yb | Fluidigm | 201173A | 1.6 | 242.284 | PBMNCs |
| Anti-human CD338 (ABCG2) (clone 5D3)-purified | surface | BD Pharmingen | cat#: 552823 | 175Lu | Fluidigm | 201175A | 0.2 | 285.508 | RPMI-MR20 cells untreated |
| Anti-human CD319 (CRACC) (clone 162.1)-purified | surface | Biolegend | cat#: 331802 | 176Yb | Fluidigm | 201176A | 0.8 | 331.251 | PBMNCs |

Supplementary Table 2: **Antibodies and metals used in the study cohort.** List of surface and intracellular antibodies used in our designed panels and respective metals (with providers and identifier). In labeled antibodies, we determined the metal content per antibody, evaluated the efficacy using indicated positive controls, and listed the optimal concentration used in CyTOF analysis.

| SOFTWARE AND ALGORITHMS | SOURCE | IDENTIFIER |
|---|---|---|
| Cytobank | Kotecha N. et al., 2010 | https://www.cytobank.org |
| Concatenation tool | Cytobank | https://support.cytobank.org/hc/en-us/articles/206336147-FCS-file-concatenation-tool |
| Normalizer | Finck R. et al., 2013 | https://github.com/nolanlab/bead-normalization/releases |
| SPADE | Cytobank | https://www.cytobank.org |
| R | R Core Team, 2016 | https://www.R-project.org/ |
| SPADE (R package) | Linderman MD. et al., 2016<br>Linderman MD. et al., 2012<br>Qiu P. et al., 2011<br>Bendal SC. Et al., 2011<br>Linderman MD. et al., 2010 | https://github.com/nolanlab/spade |
| shiny (R package) | Chang W. et al., 2019 | https://CRAN.R-project.org/package=shiny |
| shinyBS (R package) | Bailey E., et al., 2015 | https://CRAN.R-project.org/package=shinyBS |
| igraph (R package) | Csardi G. and Nepusz T., 2006 | http://igraph.org |
| ggplot2 | Wickham H., 2016 | https://ggplot2.tidyverse.org. |
| Cairo (R package) | Urbanek S. and Horner J., 2019 | https://CRAN.R-project.org/package=Cairo |
| gplots (R package) | Warnes GR. et al., 2019 | https://CRAN.R-project.org/package=gplots |
| colorRamps (R package) | Keitt T., 2012 | https://CRAN.R-project.org/package=colorRamps |
| plotly | Sievert C., 2018 | https://plotly-r.com |
| DT (R package) | Xie Y., 2019 | https://CRAN.R-project.org/package=DT |
| SPADEVizR (R package) | Gautreau G., et al., 2017 | https://github.com/tchitchek-lab/SPADEVizR |
| shinydashboard (R package) | Chang W. and Borges Ribeiro B., 2018 | https://CRAN.R-project.org/package=shinydashboard |
| htmlwidgets (R package) | Vaidyanathan R. et al., 2019 | https://CRAN.R-project.org/package=htmlwidgets |
| openxlsx (R package) | Schauberger P. and Walker A., 2019 | https://CRAN.R-project.org/package=openxlsx |
| queryBuilder | Lieberman H. and Sorel D. | https://github.com/harveyl888/queryBuilder |
| rhtmlHeatmap | Displayr | https://github.com/Displayr/rhtmlHeatmap |
| RColorBrewer (R package) | Neuwirth E., 2014 | https://CRAN.R-project.org/package=RColorBrewer |
| viridisLite (R package) | Garnier S., 2018 | https://CRAN.R-project.org/package=viridisLite |
| grid | R Core Team, 2016 | https://www.R-project.org/ |
| gridExtra (R package) | Auguie B., 2017 | https://CRAN.R-project.org/package=gridExtra |
| circlize | Gu Z. et al., 2014 | https://github.com/jokergoo/circlize |
| ComplexHeatmap | Gu Z. et al., 2016 | https://github.com/jokergoo/ComplexHeatmap |
| VennDiagram (R package) | Chen H., 2018 | https://CRAN.R-project.org/package=VennDiagram |
| FactoMineR (R package) | Le S. et al., 2008 | https://CRAN.R-project.org/package=FactoMineR |
| factoextra (R package) | Kassambara A. and Mundt F., 2017 | https://CRAN.R-project.org/package=factoextra |
| survminer (R package) | Kassambara A. et al., 2019 | https://CRAN.R-project.org/package=survminer |
| survival (R package) | Therneau TM. and Grambsch PM., 2000 | https://CRAN.R-project.org/package=survival |
| ggcorrplot (R package) | Kassambara A., 2019 | https://CRAN.R-project.org/package=ggcorrplot |

Supplementary Table 3: **Software and algorithms used in the study.** List of software and R packages, with source and identifier of CyTOF analyzed data.

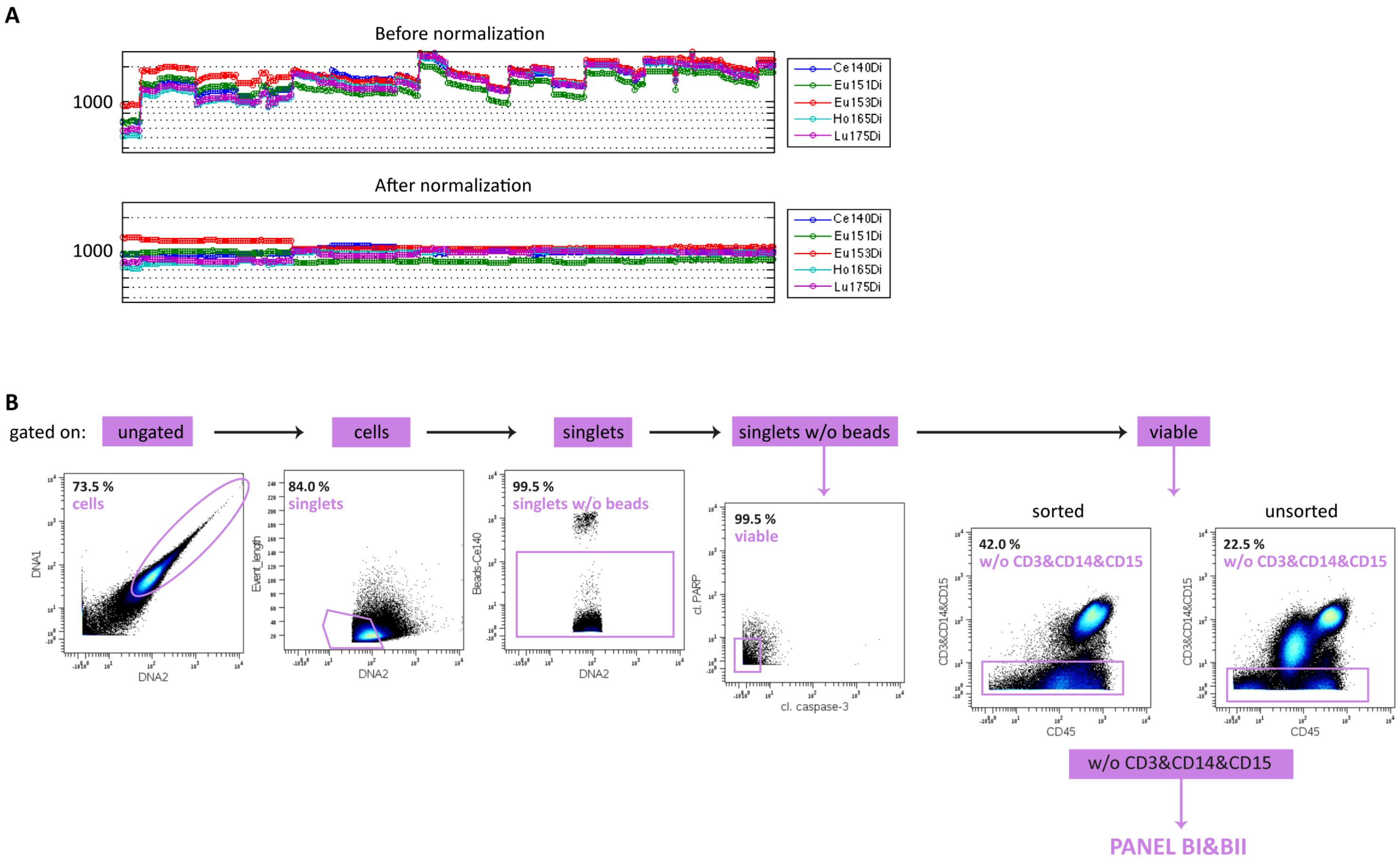


Supplementary Figure S1: **Study cohort normalization and the hierarchy of manual gating in BM samples.** (A) Graphs showing all data from our study cohort before (upper) and after (lower) normalization using Normalizer tool with EQ Four Calibration Beads (Ce140, Eu151, Eu153, Ho165 and Lu175) to correct signal fluctuation across each experiment and all batch analyses. (B) Gating strategy based on the expression of biaxial markers 191Ir (DNA1) and 193Ir (DNA2) to define cells, then gated on singlets to remove doublets followed by gating to remove beads, and finally gating on viable cells, based on lack of expression of cleaved caspase-3 and cleaved PARP (in both CyTOF panels; BI and BII panels). In panels BI and BII, final gate was defined to either remove CD3+/CD14+/CD15+ cells in unsorted samples or CD3+/CD14+ cells in CD15- sorted BM samples.

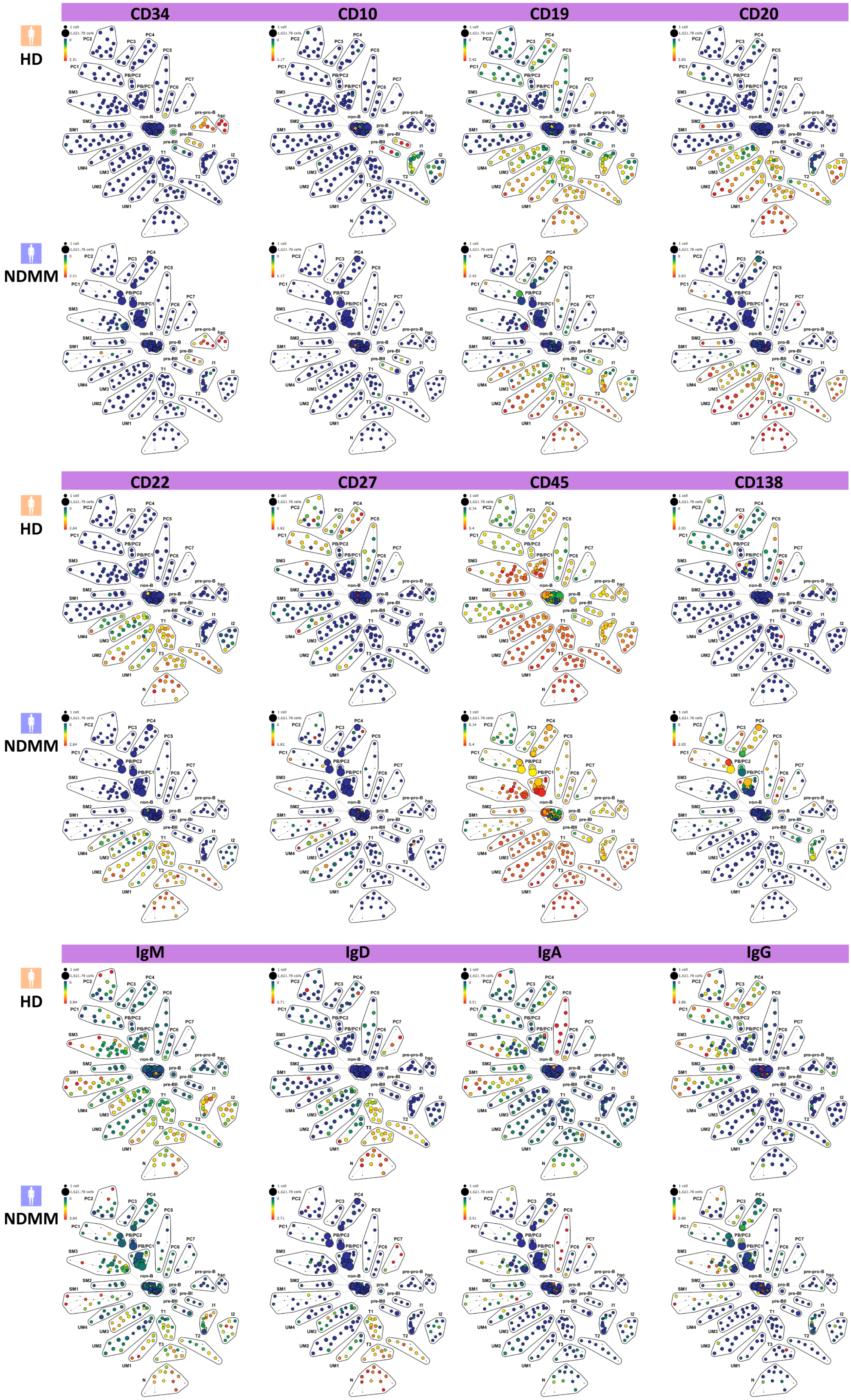
CD34
CD10
CD19
CD20
CD22
CD27
CD45
CD138
IgM
IgD
IgA
IgG
HD
NDMM
1 cell
1,621.78 cells
PC1
PC2
PC3
PC4
PC5
PC6
PC7
PB/PC1
PB/PC2
SM1
SM2
SM3
non-B
pro-B
pre-BI
pre-BII
pre-pro-B
hsc
I1
I2
T1
T2
T3
UM1
UM2
UM3
UM4
N

Supplementary Figure S2: **SPADE analysis of B lymphoid lineage cells in NDMM and HD.** SPADE analysis of cell clusters of B lymphoid lineage identified by expression of B cell markers (CD34, CD10, CD19, CD20, CD22, CD27, CD45, CD138, IgM, IgD, IgA, and IgG) in representative BM sample of HD (upper) and NDMM patient (lower). Color of each node represents the median expression of the specific clustering B cell marker (top) and the size of each node represents the amount of cells in the SPADE tree.

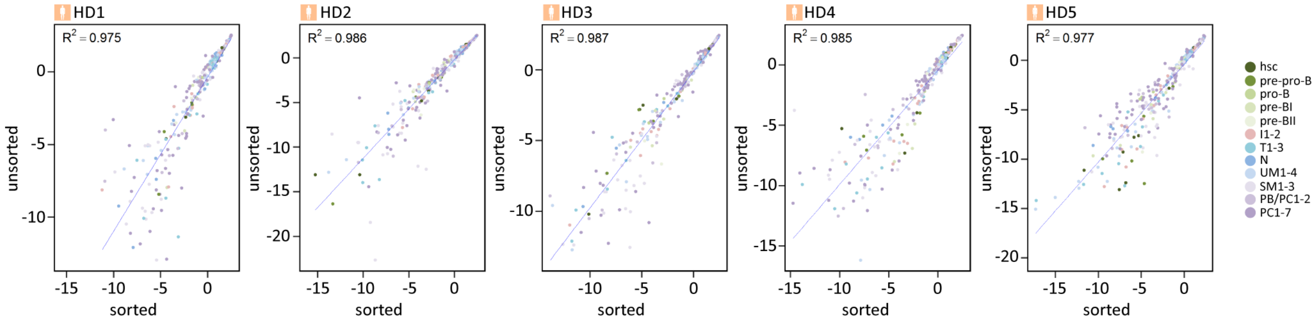


Supplementary Figure S3: **Correlation comparison of sorted and unsorted HD samples.** Scatterplots show correlation between sorted (n = 5) and unsorted (n = 5) BM samples of HD for the 13 common clustering markers (CD10, CD19, CD20, CD22, CD27, CD34, CD38, CD45, CD138, IgA, IgD, IgG, and IgM) in all B cell clusters analyzed by CyTOF and assessed by linear regression model. Each node represents the median expression of the clustering marker by mass intensity on a specific cluster (color coded); the linear model depicts the relationship by blue line; and $R^2$ is the coefficient of determination ($R^2 = 0.975$-$0.987$).

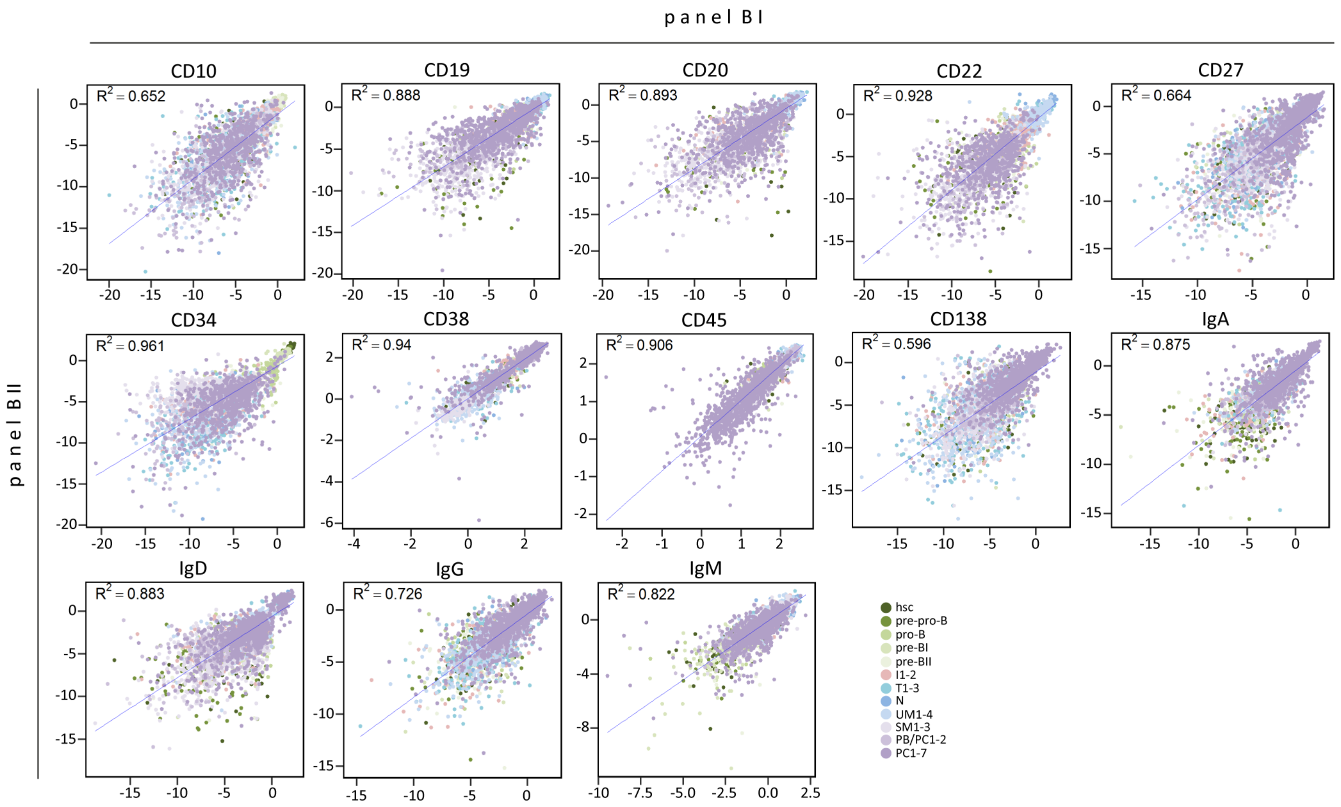


Supplementary Figure S4: **Comparison of BI panel and BII panel.** Scatterplots show correlation between BI panel and BII panel in median expression of the 13 clustering B cell markers (CD10, CD19, CD20, CD22, CD27, CD34, CD38, CD45, CD138, IgA, IgD, IgG, and IgM) for all BM samples (MGUS, SMM, NDMM, RRMM, and HD) on specific B cell clusters (color coded) and assessed by linear regression model. For each relationship, the coefficient of determination $R^2$ and the linear models are indicated (blue line).

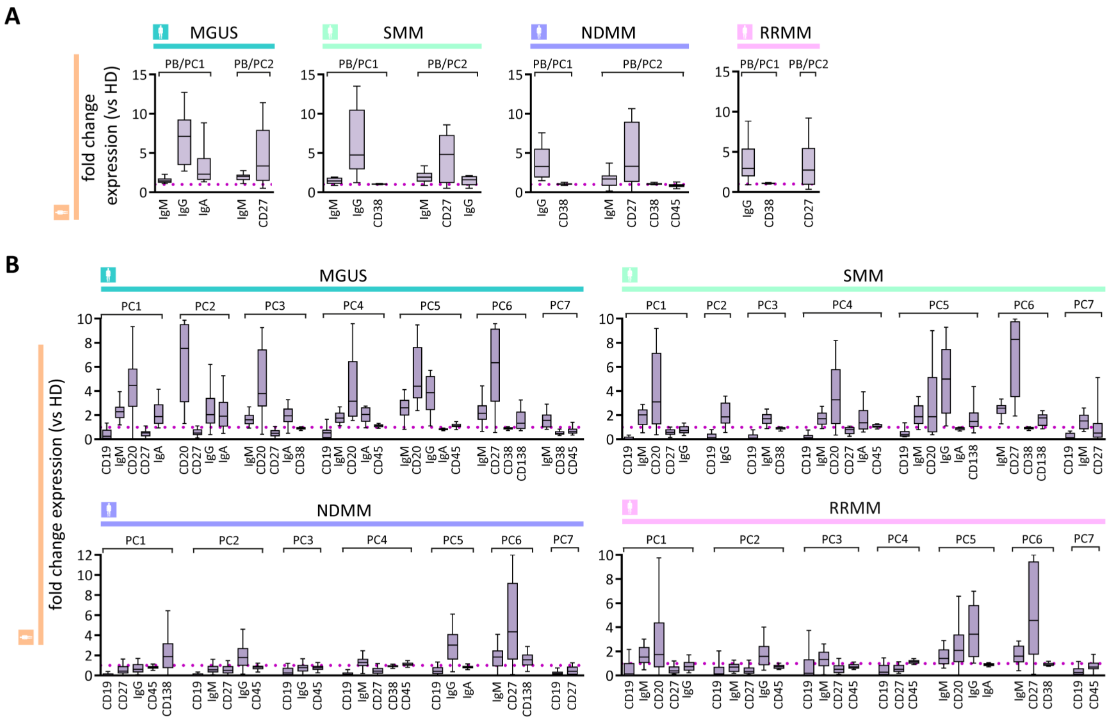


Supplementary Figure S5: **Phenotypic expression of B cell markers in MGUS, SMM, NDMM, RRMM *versus* HD on PC clusters.** Notched boxes represent the 25th and 75th percentile values with Tukey whiskers of the ratio of statistically significant median expression for the indicated B cell markers specifically expressed on (A) plasmablasts/plasma cells (PB/PC1-2 clusters) and (B) plasma cells (PC1-7 clusters) in MGUS (n = 16), SMM (n = 25), NDMM (n = 43), and RRMM (n = 104) versus HD (n = 10) by Mann-Whitney U test ($p < 0.05$).

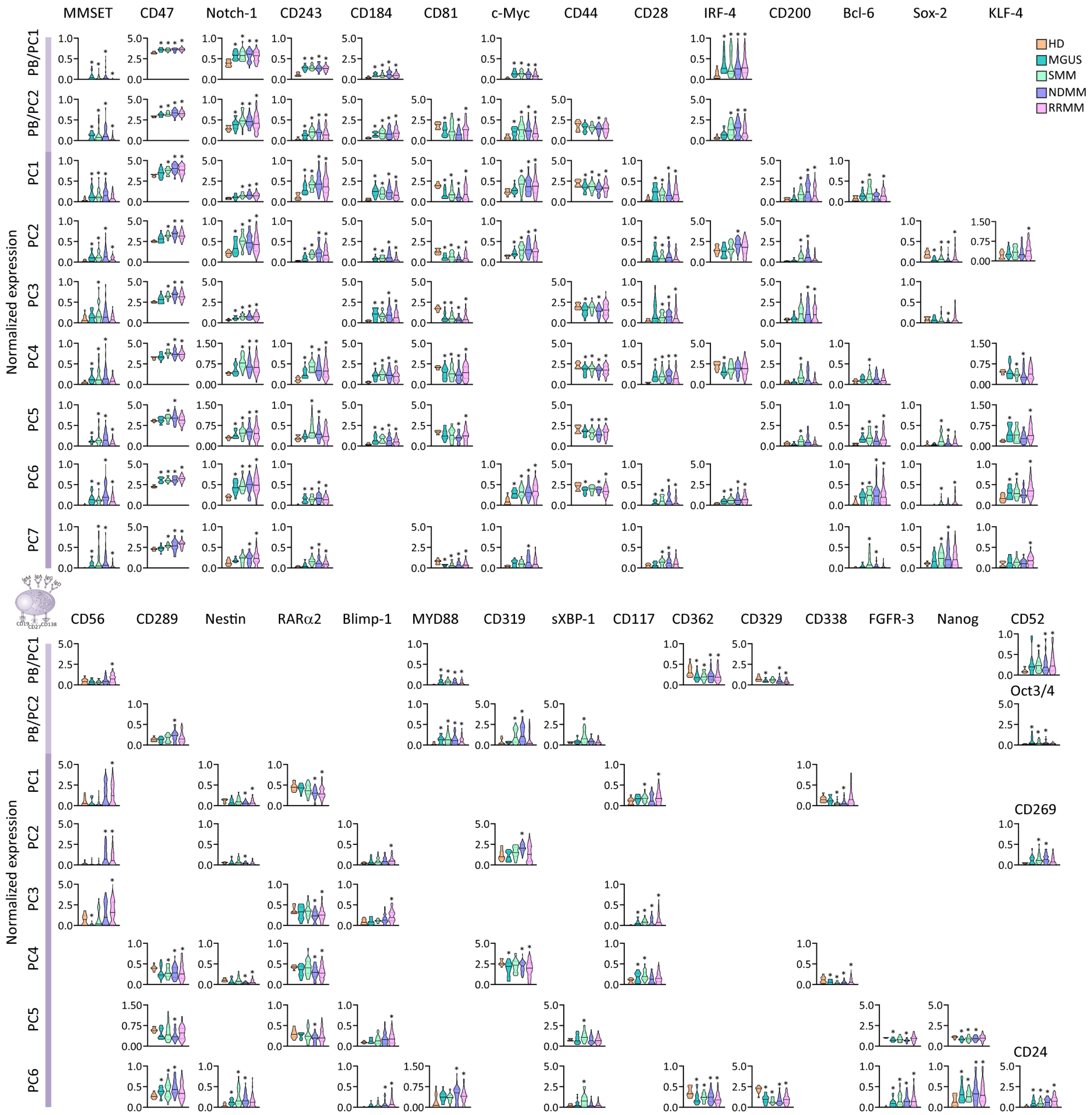


Supplementary Figure S6: **Significant normalized expression of BI and BII signaling markers in PC clusters.** Violin plots show statistically significant normalized median expression of signaling markers in plasmablast/plasma cell clusters (PB/PC1-2) and plasma cell clusters (PC1-7) in MGUS (n = 16), SMM (n = 25), NDMM (n = 43), and RRMM (n = 104) versus HD (n = 10) represented by color code. Significant differences between disease stage and HD are defined by Dunn's multiple comparison test after the Kruskal-Wallis one-way analysis of variance by ranks test, *p value < 0.05.

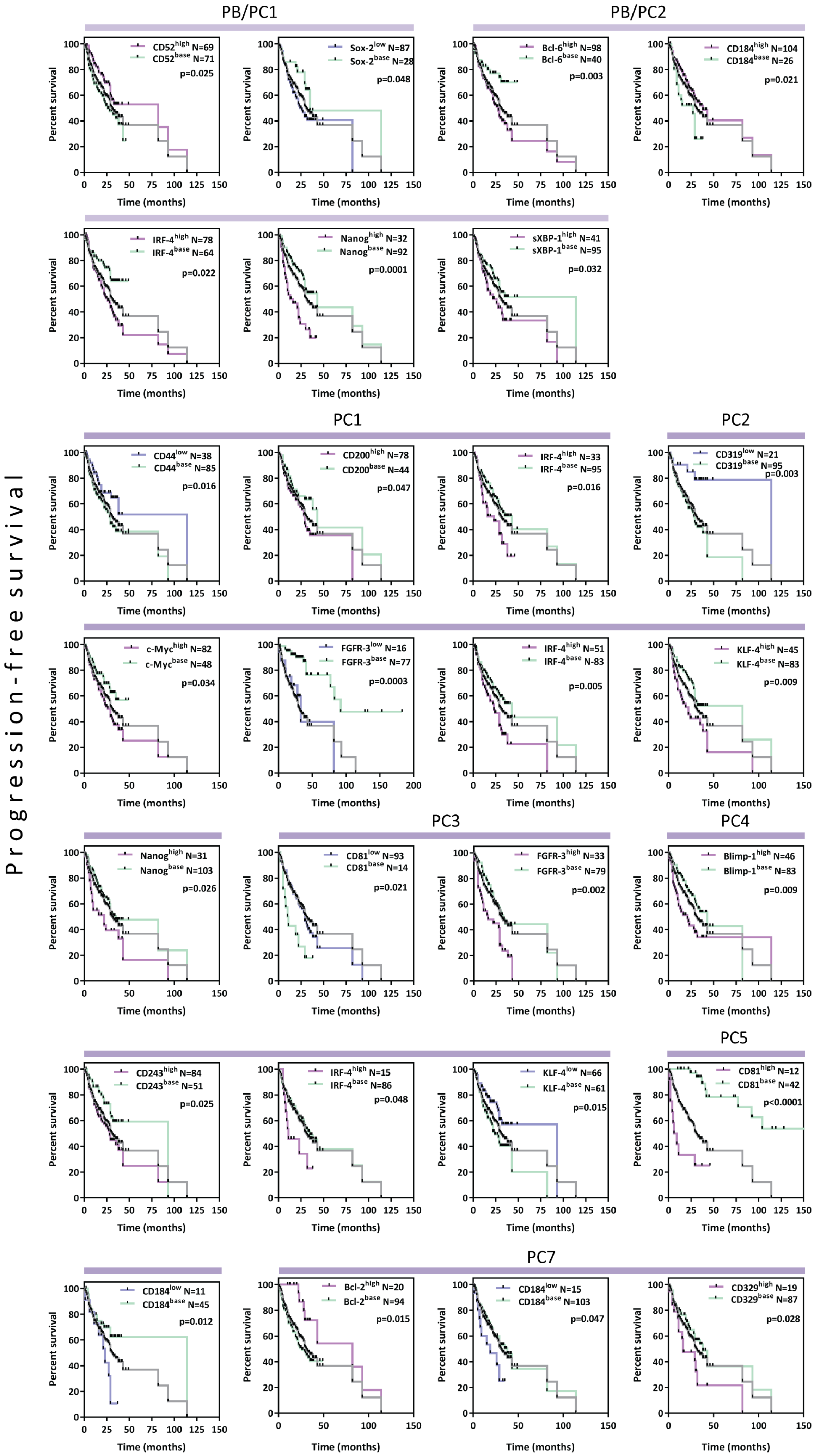
Progression-free survival
PB/PC1
PB/PC2
CD52high N=69
CD52base N=71
p=0.025
Sox-2low N=87
Sox-2base N=28
p=0.048
Bcl-6high N=98
Bcl-6base N=40
p=0.003
CD184high N=104
CD184base N=26
p=0.021
IRF-4high N=78
IRF-4base N=64
p=0.022
Nanoghigh N=32
Nanogbase N=92
p=0.0001
sXBP-1high N=41
sXBP-1base N=95
p=0.032
PC1
PC2
CD44low N=38
CD44base N=85
p=0.016
CD200high N=78
CD200base N=44
p=0.047
IRF-4high N=33
IRF-4base N=95
p=0.016
CD319low N=21
CD319base N=95
p=0.003
c-Mychigh N=82
c-Mycbase N=48
p=0.034
FGFR-3low N=16
FGFR-3base N=77
p=0.0003
IRF-4high N=51
IRF-4base N=83
p=0.005
KLF-4high N=45
KLF-4base N=83
p=0.009
PC3
PC4
Nanoghigh N=31
Nanogbase N=103
p=0.026
CD81low N=93
CD81base N=14
p=0.021
FGFR-3high N=33
FGFR-3base N=79
p=0.002
Blimp-1high N=46
Blimp-1base N=83
p=0.009
PC5
CD243high N=84
CD243base N=51
p=0.025
IRF-4high N=15
IRF-4base N=86
p=0.048
KLF-4low N=66
KLF-4base N=61
p=0.015
CD81high N=12
CD81base N=42
p<0.0001
PC7
CD184low N=11
CD184base N=45
p=0.012
Bcl-2high N=20
Bcl-2base N=94
p=0.015
CD184low N=15
CD184base N=103
p=0.047
CD329high N=19
CD329base N=87
p=0.028
Percent survival
Time (months)

Supplementary Figure S7: **MM patients' progression free survival correlated with statistically significant aberrant expression level of signaling markers.** Kaplan-Meier analyses of MM patients' progression free survival according to aberrant expression level of signaling markers (CD52, MYD88, Sox-2, Bcl-6, CD184, IRF-4, Nanog, sXBP-1, CD44, CD200, CD319, c-Myc, FGFR-3, KLF-4, CD81, Blimp-1, CD243, Bcl-2 and CD329) different from HD; red curve is higher than HD or purple curve is lower than HD *versus* patients with baseline values similar to HD (green curve, base). There were statistically significant differences in survival correlated in plasmablast/plasma cell clusters (PB/PC1-2) and plasma cell clusters (PC1-5,7), represented by color code and calculated by Kaplan-Meier log-rank test (*p value < 0.05, n = 159 gray line).
